# Protein Phosphatases Type 2C Group A Interact with and Regulate the Stability of ACC Synthase 7 in Arabidopsis

**DOI:** 10.3390/cells9040978

**Published:** 2020-04-15

**Authors:** Małgorzata Marczak, Agata Cieśla, Maciej Janicki, Anna Kasprowicz-Maluśki, Piotr Kubiak, Agnieszka Ludwików

**Affiliations:** 1 Laboratory of Biotechnology, Institute of Molecular Biology and Biotechnology, Faculty of Biology, Adam Mickiewicz University in Poznan, Uniwersytetu Poznańskiego 6, 61-614 Poznań, Poland; mm59976@amu.edu.pl (M.M.); agacie3@amu.edu.pl (A.C.); mj39518@amu.edu.pl (M.J.); 2 Department of Molecular and Cellular Biology, Institute of Molecular Biology and Biotechnology, Faculty of Biology, Adam Mickiewicz University in Poznan, Uniwersytetu Poznańskiego 6, 61-614 Poznań, Poland; akas@amu.edu.pl; 3 Department of Biotechnology and Food Microbiology, University of Life Sciences, Wojska Polskiego 48, 60-627 Poznań, Poland; piotr.kubiak@up.poznan.pl

**Keywords:** ABA signaling, ethylene biosynthesis, ACC synthase, protein modeling, ubiquitin–proteasome system, mcBiFC–FRET–FLIM, *Arabidopsis thaliana*

## Abstract

Ethylene is an important plant hormone that controls growth, development, aging and stress responses. The rate-limiting enzymes in ethylene biosynthesis, the 1-aminocyclopropane-1-carboxylate synthases (ACSs), are strictly regulated at many levels, including posttranslational control of protein half-life. Reversible phosphorylation/dephosphorylation events play a pivotal role as signals for ubiquitin-dependent degradation. We showed previously that ABI1, a group A protein phosphatase type 2C (PP2C) and a key negative regulator of abscisic acid signaling regulates type I ACS stability. Here we provide evidence that ABI1 also contributes to the regulation of ethylene biosynthesis via ACS7, a type III ACS without known regulatory domains. Using various approaches, we show that ACS7 interacts with ABI1, ABI2 and HAB1. We use molecular modeling to predict the amino acid residues involved in ABI1/ACS7 complex formation and confirm these predictions by mcBiFC–FRET–FLIM analysis. Using a cell-free degradation assay, we show that proteasomal degradation of ACS7 is delayed in protein extracts prepared from PP2C type A knockout plants, compared to a wild-type extract. This study therefore shows that ACS7 undergoes complex regulation governed by ABI1, ABI2 and HAB1. Furthermore, this suggests that ACS7, together with PP2Cs, plays an essential role in maintaining appropriate levels of ethylene in Arabidopsis.

## 1. Introduction

The plant hormone ethylene affects many physiological and agronomic traits in plants, with both positive and negative effects on plant growth and development: It promotes seed germination, respiration, ripening, senescence and abscission [1,2,3,4]. On the other hand, the increased ethylene synthesis that occurs under stress conditions has detrimental effects on plant vitality and survival [5,6]. Thus, an understanding of ethylene action and synthesis will provide tools by which to regulate ethylene content and manipulate specific plant responses.

Ethylene biosynthesis is well-characterized and consists of two relatively simple reactions. In the first step, S-adenosyl methionine is catalyzed by 1-aminocyclopropane 1-carboxylate synthase (ACS; also abbreviated as ACC synthase) to 1-aminocyclopropane 1-carboxylate (ACC). In the second step, ACC is converted to ethylene with release of carbon dioxide and cyanide by 1-aminocyclopropane 1-carboxylate oxidase (ACO). ACS is thus a crucial enzyme in ethylene biosynthesis and in all plant species is encoded by a multigene family consisting of nine members in Lycopersicon esculentum, six in *Oriza sativa* and 11 in poplar [7,8]. In Arabidopsis, the ACS gene family comprises 12 members, including eight genes encoding functional enzymes (ACS2, ACS4-9 and ACS11), a single inactive form of ACS1 and a pseudogene ACS3 [9]. 

The available domain structures of the ACS isozymes allow their classification based on the presence or absence of non-catalytic C-terminal phosphorylation motifs [10]. Type I ACSs contain a C-terminal fragment that includes phosphorylation sites for both mitogen-activated protein kinases (MAPKs) and calcium-dependent protein kinases (CDPKs). In *Arabidopsis thaliana*, ACS2 and ACS6 belong to type I. Type II isozymes carry only the CDPK target site, and type III isozymes lack both MAPK and CDPK sites [11,12]. In Arabidopsis, ACS4, ACS5, ACS8, ACS9 and ACS11 are type II ACSs, while there is only a single type III isozyme, ACS7.

Although ACS7 lacks known regulatory domains, it still undergoes ubiquitin-dependent proteasomal degradation. The E3 ubiquitin ligase, XBAT32, initiates this process by attachment of ubiquitin to ACS7 [13,14]. XBAT32 is member of the RING domain-containing ankyrin repeat E3 ligase subfamily and was first characterized as a positive regulator of lateral root development. Knockout plants (*xbat32*) exhibit ethylene-related phenotypes, such as a reduced number of lateral roots or an elevated ethylene level [15]. Yeast two-hybrid analysis showed that XBAT32 interacts with ACS4 and ACS7 (type II and type III isoforms, respectively) and is able to attach ubiquitin to both synthases in an in vitro assay [13]. Further in vitro and *in planta* experiments confirmed that XBAT32 directs ACS7 for proteasomal degradation [14]. These workers used a cell-free assay to show that degradation of His-Flag-ACS7 can be inhibited by MG132, a 26S proteasome inhibitor, and then they performed *in planta* experiments, to demonstrate that HA–ACS7 expressed in a *xbat32-1* mutant strain (*35S:HA–ACS7/xbat32-1*) is much more stable than when expressed in wild-type plants. These results confirm that ACS7 degradation is XBAT32 dependent [14].

Proteasome-dependent degradation of ACS7 implies that, as for type I and II ACSs, phosphorylation/dephosphorylation events control the stability and activity of ACS7. ACS7 was found to interact with a 14-3-3 protein, a category of regulatory protein involved in a variety of biological processes [16]; for example, in mammalian cells, a 14-3-3 protein protects p53 (a short-lived tumor suppressor protein) from proteasomal degradation [17]. ACSs were co-purified with a 14-3-3 protein during proteomic characterization of 14-3-3 protein complexes [16], and the 14-3-3ω isoform was later shown to interact with ACS7 and increase its stability [18]. As the 14-3-3 proteins mainly interact with phosphorylated proteins, this suggests that ACS7 undergoes phosphorylation. Indeed, ACS7 can be phosphorylated by the calcium-dependent protein kinase AtCDPK16 in vitro [19]. AtCDPK16 can phosphorylate three different residues, S216, T296 and S299, within the ACS7 sequence. What is more, phosphorylated ACS7 is more catalytically active than non-phosphorylated ACS7. This suggests that CDPK16 can enhance ACS7 activity, but these in vitro results need confirmation in cells or plants [19].

Xiong and co-workers performed a sequence alignment of N-terminal and C-terminal fragments of all three types of ACS from different species [20]. They noticed that type III ACSs have a longer N-terminal region than type I and II. An in vivo degradation assay showed that truncated ACS7 lacking the first 14 N-terminal amino acids has a longer half-life than a full-length version in light-grown plants. However, this phenomenon was not observed in etiolated seedlings, when both versions of ACS7 were stable, in line with the finding that light inhibits ethylene production [21]. Moreover, the 14 N-terminal amino acids of ACS7 are crucial for sensing senescence signals during leaf development: N-terminal ACS7 degradation is negatively regulated by leaf senescence [22].

Expression of the *ACS7* gene depends on both the developmental stage of the plant and organ type. In mature plants, *ACS7* is mainly expressed in roots and young leaves [23]. However, analysis of *acs7* plants revealed that this mutant is hypersensitive to exogenous ABA and, in addition, has elevated endogenous ABA levels during salt stress. These surprising findings suggest that ACS7 may function as a negative regulator of ABA signaling during abiotic-stress-induced ethylene biosynthesis [24].

In our previous publication we demonstrated that the PP2C ABI1 interacts with ACS6, a type I ACS [25]. Because in *A. thaliana* there is only a single type III ACS, i.e., ACS7, we were interested to learn whether group A PP2Cs, as negative regulators of ABA signaling, can affect the function of this protein. We found that group A PP2Cs, including ABI1, ABI2, and HAB1, interact with ACS7 and are important for the regulation of ACS7 protein turnover. Molecular modeling of the ACS7-ABI1 interaction revealed characteristic structural residues in the ABI1 sequence that are essential for formation of the complex, including the W300 residue of ABI1. In addition, in a cell-free degradation assay, we demonstrated that ACS7 turnover is significantly affected by ABI1, ABI2 and HAB1. Overexpression of ACS7 increases the level of ethylene in *A. thaliana* in both WT and *abi1td* backgrounds in response to MG132 treatment. We conclude that A PP2Cs are a group of precise and specific regulators of ethylene biosynthesis.

## 2. Materials and Methods

### 2.1. Plant Materials, Growth Conditions and Treatments

*Arabidopsis thaliana* WT Columbia (Col-0) plants and the *abi1td* (SALK_076309), *abi2-2* (SALK_015166) and *hab1-1* mutants (SALK_002104) were used in the study [26,27]. Seed sterilization and plant growth were performed as described [25]. For cell-free degradation assays 7-day-old seedlings were treated with 100 µM MG132 (Sigma-Aldrich, Poznan, Polandas appropriate. For ethylene measurement 2-week-old seedlings of WT Col-0, *abi1td* and transgenic plants were treated with 100 µM MG132 or equivalent mock control for 24 h.

### 2.2. Plant Transformation

The *ACS7* coding sequence was introduced into pEarlyGate 103 plant transformation vector using Gateway technology (Invitrogen, ThermoFisher Scientific, Warsaw, Poland) and then transformed into *Agrobacterium tumefaciens* (strain C58C1) [28]. Next, the transgene was introduced into Arabidopsis wild type Col-0 and *abi1td* mutant, using the floral dip method [29]. Transgenic *35S*:*His–GFP–ACS7*/Col-0 and *35S*:*His–GFP–ACS7*/*abi1td* T1 plants were selected on solid MS media supplemented with 50 µM DL-phosphinothricin (Sigma-Aldrich, Poznan, Poland). Plants showing herbicide resistance were transferred to soil, and the presence of the transgene was determined by PCR, using primers specific for GFP, and by Western blot, using an antibody against GFP (Santa Cruz Biotechnology, Dallas, TX, USA). Transgenic plants for subsequent analysis were selected from the T3 generation.

### 2.3. Vector Constructs 

For expression, subcellular localization and interaction analysis of ACS7 protein, the full coding sequences of ACS7 and the ABI1, ABI2 and HAB1 PP2Cs were PCR-amplified by using specific primer pairs (Supplementary Table S1) and cloned into the pENTR/SD/D-TOPO vector (Invitrogen). To introduce point mutations into the ABI1 sequence (yielding ABI1 W300A), a PCR reaction was conducted, with a given primer (Supplementary Table S1*)* and a specific entry clone as a template (pENTR vector), using the QuikChange II XL Site-Directed Mutagenesis Kit (Agilent, Santa Clara, CA, USA), according to the manufacturer’s protocol. The presence of each mutation was confirmed by sequencing. To generate destination vectors expressing N- or C-terminal protein fusions, full pENTR constructs were digested with *Mlu*I (ThermoFisher Scientific, Warsaw, Poland), and the resulting fragments were recombined with pDEST™15, pDEST™17 (ThermoFisher Scientific, Warsaw, Poland), pEarlyGate103, pSITE4CA, pSITE2CA [30], modified pSAT3-cCFP-DEST and the pSAT5-DEST-nVenus [31], using Gateway^®^LR *Clonase*^®^ II Enzyme Mix (Invitrogen, ThermoFisher Scientific, Warsaw, Poland). Final constructs were verified by restriction digestion and sequencing.

### 2.4. Microscopy Studies in Arabidopsis Protoplasts

Leaf mesophyll protoplasts were isolated from 3-week-old *A. thaliana* Col-0 plants and transformed as reported in Ludwikow et al. and Mituła et al. [25,27,31]. For protein subcellular localization studies, protoplasts, transformed with GFP- or mRFP-tagged constructs, were analyzed by using a Nikon A1R confocal microscope. Protoplasts were observed with Plan Apo × 20/40. The eGFP fluorescence was excited with a 488 nm laser and captured by a 500–550 nm emission filter, while mRFP was excited with a 561 nm laser and captured by using a 570–620 nm emission filter. Data were prepared in Nikon NIS-Elements and ImageJ MBF software. Colocalization was quantified as Pearson’s correlation coefficient for ten independent protoplasts or for magnified selected images with Nikon NIS-Elements.

For multicolor bimolecular fluorescence complementation (mcBiFC) analysis, various combinations of plasmids encoding cCFP and nVenus fusion proteins were mixed in a 1:1 (*w*/*w*) ratio, and 12 μg of the mixture of plasmid DNA was used for PEG-mediated transformation of 100 µL protoplast suspension. After transformation, protoplasts were incubated overnight at room temperature. Plant protoplasts were viewed directly under a Nikon A1R confocal laser-scanning microscope equipped with a 488 nm argon laser line; images were captured by 500–550 nm emission filter. 

Förster resonance energy transfer (FRET) and fluorescence lifetime imaging (FLIM) were performed as follows. FLIM was prepared by using the Picoquant PicoHarp TCSPC Module with a Nikon A1R confocal microscope. The above-described BiFC complex nVenus–cCFP and mRFP proteins were the donor: acceptor pair, respectively. The donor was excited with a 485 nm pulsed diode laser (PDL 800-D; 40 mHz). The excitation light was directly coupled into the microscope. Photons were detected, using a SPAD detector module. The fluorescence of the reconstructed homodimer nVenus–ACS7 and cCFP–ACS7 complex was selected, using a 500/550 nm filter. Data were prepared, using Picoquant’s Symphotime software. From the intensity images, complete fluorescence lifetime decays were calculated per pixel for whole images, as well as for selected image fragments. For FLIM analysis, the fluorescence lifetime of one component was fixed to the value found for the native donor (T_D_). A χ^2^ of 1 was considered a perfect fit. The FRET efficiency (E) was calculated as E = 1 − τ_DA_/τ_D_ × 100%, where τ_D_ is the fluorescence lifetime of the donor in the absence of acceptor, and τ_DA_ that of the donor in the presence of acceptor [32,33].

Statistical analysis of the results was performed for transformed protoplasts (*n* > 10 for each sample), and Student’s *t*-test was performed to determine the statistical significance of differences between samples. Error bars represent standard deviation (SD).

### 2.5. Ethylene Measurements

Ethylene was quantified by gas chromatography. An Agilent 7890A (Agilent Technologies, Santa Clara, CA, USA) chromatograph equipped with a Carboxen^®^-1006 PLOT column (30 m × 0.53 mm × 30 µm; Supelco, USA) was used. A sample of 1 mL was drawn from the headspace of a vial with an A-2 series gas-tight syringe (VICI Precision Sampling, Baton Rouge, LA, USA). The sample was then injected into the S/SL inlet, operated at 120 °C, in splitless mode. The separation was performed isothermally at 120 °C, with a flow rate of 5.5 mL/min of helium as carrier gas. FID at 250 °C was used for detection. Quantification was based on peak area. A mixture of ethylene in synthetic air (Air Liquide, Paris, France) was used as standard. 

### 2.6. Recombinant Protein Expression and Purification 

Recombinant GST–ACS7, His–ABI1 and His–ABI2 fusion proteins were expressed in *E. coli* BL21-CodonPlus (DE3)-RIL competent cells and purified, using Glutathione-Sepharose 4B and Ni-Sepharose 6 Fast Flow (GE Healthcare, Chicago, IL, USA), according to manufacturer-provided protocols.

### 2.7. Pull-Down Assay

GST pull-down assays were performed as previously described in Hagemeier et al. and Mituła et al. [31,34]. In brief, GST–ACS7 protein pre-coupled to Glutathione-Sepharose 4B was preincubated with 1 mg/mL bovine serum albumin (BSA), for 10 min, at room temperature. Next, His-tagged ABI1 or ABI2 protein was added and rocked in a final volume of 100 μL EBC buffer (100 mM NaCl; 0.5% Nonidet P-40; 100 mM NaF; 200 μM sodium orthovanadate; 50 mM Tris-HCl pH 8.0), for 1 h, at room temperature. After incubation, the resin was washed three times with 1 mL NENT buffer (100 mM/200 mM/300 mM NaCl; 1 mM EDTA; 0.5% Nonidet P-40; 20 mM Tris-HCl pH 8.0), with increasing NaCl concentration. Samples were then boiled in SDS–PAGE sample buffer and analyzed by immunoblotting. 

### 2.8. Cell-Free Degradation Assay

The cell-free degradation assay was performed essentially as described in Ludwików et al. and Mituła et al. [25,31]: 500 µg cell extract was mixed with 300 µg recombinant, GST–ACS7 and, following incubation at 22 °C, GST–ACS7 protein was detected with anti-GST (1:5000; MoBiTec Gottingen, Germany). Ponceau staining or anti-actin antibody (1:4000; Merck, Darmstadt, Germany) was used as an internal control. 

### 2.9. Immunoblotting

For immunoblotting, denatured proteins were separated on a 10% SDS–PAGE gel and transferred to Immobilon P (, Merck Millipore, Burlington, VT, USA). The membranes were blocked for 1 h in PBS-T buffer, pH 7.4, containing 3%–5% blocking solution (skim milk). Membranes were washed three times, for 5 min, with PBS-T buffer, and incubated for 1 h, with anti-GST-Tag (1:5000; MoBiTec), rabbit anti-GFP (1:200; sc8334, Santa Cruz Biotechnology) or rabbit anti-His (MoBiTec). After washing, as previously done, the membranes were incubated for 1 h, with the secondary anti-rabbit antibody (1:50,000; Agrisera, Vännäs, Sweden). Detection was performed by using ECL (ThermoFisher Scientific, Warsaw, Poland), based on the manufacturer’s instructions. 

### 2.10. ACS7-ABI1 Protein Complex Modeling

A model of ACS7 (ACC synthase) protein was developed based on an advanced homology modeling protocol, which was implemented in the Schrodinger computational biology and chemistry package. Loop refinement and structure minimization of the homodimer models were done by using the Prime module (also implemented in Schrodinger). All structures were minimized, using the OPLS3 force field. To build the model of the ACS7 structure, a multi-template method was used, based on the five top-ranked homologous proteins. To construct the model of the ABI1–ACS7 complex, the Haddock webserver was used. The passive and active residues were chosen according to the mechanism of dephosphorylation. The structures obtained were clustered and ranked. Post-docking visual analysis was done, using Pymol (LIT), a graphical molecular browser, mainly to investigate the interaction sites of the two proteins in the model of the complex. Root mean square deviation (RMSD*) was calculated for the aligned complexes and calculated between $C_{\alpha }$ atoms of the targeted serine residue for the evaluated ACS7–ABI1 complexes and serine in position 175 (S175) of the predicted structure complex of SnRK2.6–ABI1, which was built based on experimental data (PDB code: 3UJG).

## 3. Results

### 3.1. ACS7 and Group A PP2Cs Are Localized in both Nucleus and Cytoplasm

**Figure 1:**
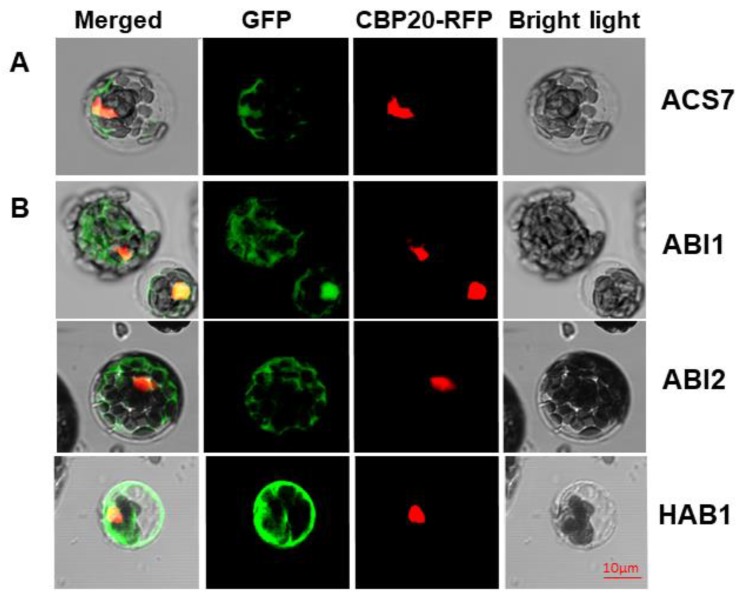
*In planta* localization study. Localization of ACS7–GFP (**A**) and type 2C phosphatases (**B**) in *Arabidopsis thaliana* protoplasts, by confocal microscopy. CBP20–RFP was used as nuclear marker. Green fluorescence (ACS7–GFP, ABI1–GFP, ABI2–GFP and HAB1–GFP) and red fluorescence (RFP–CBP20) are merged in the left-hand column with the bright field image; scale bar, 10 µm.

Before assessing the ACS7–PP2C group A interaction *in planta*, we first asked whether ACS7 and group A PP2Cs co-localize in cells. Previously, ACS7 has been localized to the cytoplasm and nucleus [19,35], while group A PP2C members, alone or complexed with interacting proteins, are predicted to occupy various compartments, including the nucleus, cytoplasm and cell membrane [25,36,37,38,39]. Accordingly, the PP2C HAB1 localizes to the nucleus and cytoplasm [37], whereas another PP2C, ABI1, was identified in the nucleus and in association with the plasma membrane [36]. To investigate putative ACS7 interaction partners, we prepared N-terminal fusions of ACS7 and PP2C group A (ABI1, ABI2 and HAB1) proteins with green fluorescent protein (GFP). All constructs were co-transformed into Arabidopsis protoplasts, together with the nuclear marker CBP20–RFP [40]. As seen in Figure 1, ACS7–GFP, ABI1–GFP, ABI2–GFP and HAB1–GFP were predominantly localized in the nucleus and cytoplasm, supporting a dual localization for both types of protein (see also Figure S1). CBP20–RFP was consistently localized in the nucleus, confirming its location in that cellular compartment only. 

### 3.2. ACS7 Interacts with Group A Protein Phosphatases Type 2C

**Figure 2:**
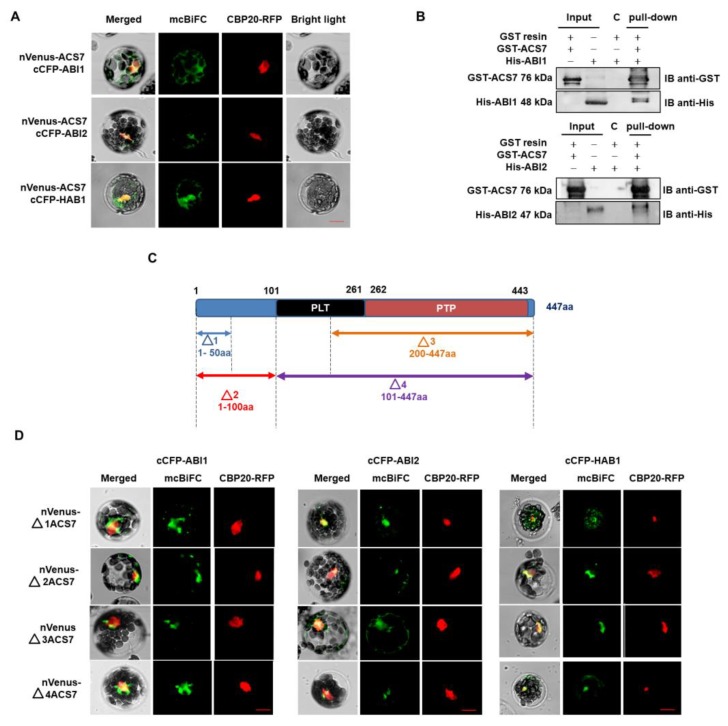
ACS7 interacts with type 2C protein phosphatases. (**A**) ACS7 interacts with group A PP2C proteins. ACS7 was fused with the N-terminal fragment of Venus protein, while type C phosphatases were fused with C-terminal fragment of CFP. A fluorescence signal was observed in cells co-expressing nVenus–ACS7; and cCFP–ABI1, nVenus–ACS7 and cCFP–ABI2; and nVenus–ACS7 and cCFP–HAB1, in the cytoplasm and nucleus. Each transformation control also included a plasmid encoding CBP20 (nuclear cap-binding protein subunit 2) fused with a full-length fluorescent protein with distinct spectral characteristics (mRFP). In addition, CBP20–RFP served as a nuclear marker; scale bar, 10 µm; (**B**) GST–ACS7 protein was pre-coupled to the resin and incubated for 1 h, with His–ABI1 and His–ABI2 individually. After washing, samples were separated on 10% SDS–PAGE gels, transferred to PVDF membrane and analyzed by Western blot, with anti-GST and anti-His antibodies. All two PP2Cs were able to interact with GST–ACS7. Lines with input represent half of the recombinant protein used for pull-down. C-represent control where His-tagged protein was incubated with GST resin and washed the same way as pull-down sample. The results shown are representative of at least two independent experiments; (**C**) schematic representation of the ACS7 domain structure and ACS7 deletion variants (N-terminal: Δ1 1-50 aa, Δ2 1-100 aa, and C-terminal Δ3 200-447 aa and Δ4 101-447 aa). The ACS7 Δ2 variant contains the pyridoxal- and phosphate-dependent transferase domain. The C-terminal deletion form of ACS7 Δ3, includes pyridoxal phosphatase-dependent transferase subdomain 1. ACS7 Δ4 includes both subdomains (PLT and PTP); PLT = pyridoxal phosphate-dependent transferase, major region subdomain 1; PTP = pyridoxal phosphate-dependent transferase, major region, subdomain 2; (**D**) BiFC analysis showing interaction between ABI1, ABI2 and HAB1 PP2Cs and ACS7 deletion forms. CBP20 fused with RFP was used as nuclear marker. Deletion forms of ACS7 were fused with nVenus (nVenus–ACS7), while phosphatases were fused with cCFP (cCFP–PP2C); scale bar, 10 µm.

Since co-localization studies gave promising results, we investigated whether ACS7 and group A PP2Cs interact in living *A. thaliana* cells, using multicolor bimolecular fluorescence complementation (mcBiFC) [41]. The mcBiFC approach involves the reconstruction of a fluorescent complex when two proteins fused to non-fluorescent fragments of a fluorescent protein interact with each other. Thus, *A. thaliana* was co-transformed with ACS7 fused with the N-terminal part of Venus (nVenus) protein and type 2C phosphatases fused with the C-terminal part of CFP protein (cCFP). Simultaneous introduction of a CBP20–RFP construct served as a transformation control and nuclear marker. A BiFC signal was observed in cells co-expressing nVenus–ACS7 and either cCFP–ABI1, cCFP–ABI2 or cCFP–HAB1, showing that ACS7 interacts with all three PP2Cs. In all cases, the BiFC signal was predominant in the cytoplasm, but there was some overlap with the CBP20–RFP fluorescence, suggesting that a proportion of the ACS7–PP2C complexes enters the nucleus (Figure 2A; Figure S2).

The formation of ACS7-group A PP2C complexes was also analyzed, using an in vitro GST pull-down assay (Figure 2B). ACS7 and group A PP2C proteins were tagged with GST and His sequences, respectively, and overexpressed individually in a bacterial system. Recombinant GST–ACS7 was then incubated with His–ABI1 or His–ABI2 and GST complexes were recovered, using a GST resin. The two PP2Cs were shown to associate strongly with ACS7 in this assay.

To identify domains involved in ACS7–PP2C group A interactions, we constructed ACS7 truncations, two N-terminal (Δ1 and Δ2) and two C-terminal (Δ3 and Δ4), for mcBiFC experiments (Figure 2C). The Δ1 ACS7^1–50^ includes a short N-terminal fragment (i.e., amino acid residues 1 to 50), without any structural motifs, while the Δ2 ACS7^1–100^ fragment lacks the pyridoxal- and phosphate-dependent transferase domains. The Δ3 ACS7^200–447^ and Δ4 ACS7^101–447^ fragments include the pyridoxal phosphatase-dependent transferase subdomains (1 and 2) and pyridoxal phosphatase-dependent transferase subdomain 2, respectively. The extent of the ACS7 truncations was determined after sequence analysis of the InterPro database. All truncated ACS7 fragments were fused with the nVenus protein fragment (as a C- or N-terminal fusion) and co-transformed into protoplasts with each of the cCFP-PP2C constructs. We found that all ACS7 deletion fragments interact with the ABI1, ABI2 and HAB1 PP2Cs, demonstrating low binding specificity between these protein pairs (Figure 2D; Figure S3). This suggests that PP2Cs most likely interact at multiple sites with the ACS7 protein sequence. Taken together, the above results provide consistent support for an interaction between ACS7 and ABI1, ABI2, and HAB1.

### 3.3. Modeling of the ACS7–ABI1 PP2C Complex

**Figure 3:**
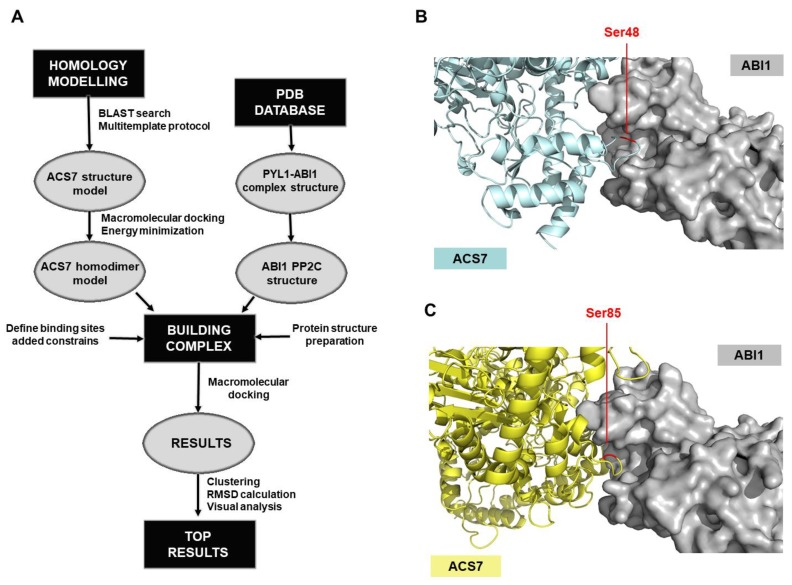
Computational procedure for obtaining the structural model of the ABI1–ACS7 complex. (**A**) The procedure for constructing the ACS7–ABI1 complex begins by obtaining the structures of the two protein components, ACS7 and ABI1. The first of these was obtained by homology modeling, while the second one was exported from the pdb database (X-ray resolved structure PYL1–ABI1 pdb code: 3KDJ). In homology modeling, a 3D full-atom protein model of a target sequence is generated based on experimental data for a related homologous protein (BLAST search). Before building the complex, the protein structures were prepared, the sites involved in the interaction were defined and constraints were added. To obtain the structures of the ABI1–ACS7 complex, a macromolecular docking algorithm (Haddock) was used. After clustering, RMSD* calculation and visual analysis, the top-ranked results for the ACS7–ABI1 complex were obtained; (**B**,**C**) docking of ACS7 residues into the structure of ABI1. Using molecular modeling, residues S48 (**B**) and S85 (**C**) of ACS7 were selected as putative ABI1 targets. The ACS7 structure is presented in blue (**B**) or yellow (**C**). The ABI1 surface is in gray. ACS7 target amino acid residues S48 and S85 in the ABI1 catalytic pocket are highlighted in red.

A model of ACS7 protein was developed, using an advanced homology modeling protocol (Figure 3). Following a BLAST search, the resolved structures of various related proteins were found. The six top-ranked structures (1B8G_A, 1M7Y_A, 1M4N_A, 1YNU_A, 1IAX_A and 3PIU_A) had > 60% sequence similarity with ACS7 with < 5% gaps; these were used as templates to build the protein structure model. Multi-templates were used to reduce the gap-sequence ratio. The protein structure was obtained by using an energy-based method, which provides a precise estimate of the stability of the binding site. After initial modeling, each loop of the ACS7 structure was refined, using the Prime module, and the whole structure was minimized in an OPLS3 force field. Based on the literature data, ACS7 forms homo- and heterodimers [23,42]. In our study, we focused on a homodimer structure, which we constructed by using a macromolecular docking algorithm (Figure 3). Thirty-two different conformers of this ACS7 homodimer were obtained, and each was clustered and minimized in the OPLS3 force field. The most stable conformer, i.e., that with the lowest potential energy (Table S2), was chosen for the next step of the workflow, to construct a model of the ACS7–ABI1 complex (Figure 3A). 

To build the model of the ACS7–ABI1 PP2C complex, the above-generated ACS7 protein model was used with an ABI1 PP2C structure imported from a previously defined ABA–PYL–ABI1 complex structure (pdb code 3KDJ) [43]. Our proposed model of ACS7–ABI1 interaction was based on a dephosphorylation reaction where the substrate binds to a specific region in the ABI1 catalytic pocket, and the phosphorylated residue loses its phosphate group. This approach allowed us to identify protein fragments relevant to the interaction. Because ABI1 PP2C is a S/T phosphatase, the binding site in ACS7 could be either a single S or T residue, both of which have hydroxyl groups that can be phosphorylated. However, after scanning for ACS7 residues accessible to ABI1, we focused on S residues, because, as described previously, these are important for the regulation of ACS protein turnover [44]. In addition, we filtered out all S residues not on the surface and those that were not predicted to be located inside loop structures. According to these criteria, amino acids S24, S48, S52, S85, S182, S183, S272 and S413 in the ACS7 sequence were selected as accessible for binding to the PP2C catalytic pocket in ABI1. 

In our working model, the ABI1 binding interface was defined as a space formed in the three-dimensional structure of the protein, containing amino acids surrounding the metal ion in the catalytic pocket (residues 125–429) (Figure 3B,C). Residues 125–429 were previously shown to play a crucial role in phosphatase activity and the mechanism of interaction with ABA receptors [45]. Before constructing the ACS7–ABI1 complex, we tested our docking model, using data from previous studies of the interaction between kinase substrate SnRK2.6 and PP2C phosphatase HAB1. In this example, serine residue S175 in SnRK2.6 was the main target of HAB1. We therefore performed a test simulation, using Haddock software, which estimates the interaction energy of protein–protein complexes, for an interaction between SnRK2.6 and ABI1, with SnRK2.6 S175 as the targeted serine residue. The metal ion in the ABI1 binding interface was not included in the Haddock simulations, however, because there are no parameters in the force field that describe metal ions. The results obtained for the resulting SnRK2.6–ABI1 complex matched well with the experimental SnRK2.6–HAB1 structure (Figure S4). For the best structure of the complex, the Haddock score was -164.7 ± 2,7, and the RMSD value was 0.9 Å (RMSD was performed for aligned complexes and calculated between the $C_{\alpha }$ atoms of targeted serine residues; Figure S5). 

**Figure 4:**
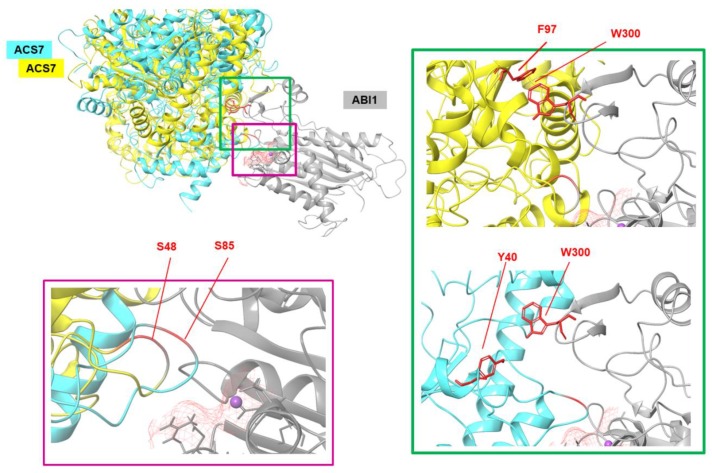
Summary model of the interactions between ACS7 and ABI1 PP2C. Zoomed-in view of the ACS7–ABI1 PP2C complex structure presented in ribbon style. Three-dimensional structures of proteins showing the binding sites and main residues involved in interactions. ABI1 PP2C with bound Mg^2+^metal ion is shown in gray. ACS7 (yellow, cyan) is presented in different orientation-based structures exposing the key S48 and S85 residues of ACS7 in the ABI1 PP2C binding pocket (left). W300 of ABI1 PP2C and F97 and Y40 of ACS7 are shown as red sticks (right).

To generate custom models of the ABI1–ACS7 complex, Haddock software was used. The above-indicated S residues of ACS7 and the ABI1 binding interface were used as constraints. The results obtained were clustered, and from each set, the geometry of the complex was selected. Based on the Haddock scoring function, all complex structures were ranked. Using these results, together with a RMSD* value (Supplementary Table S3) and visual analysis of the evaluated ACS7–PP2C complexes for each indicated target serine residue of ACS7, the residues with highest binding potency were selected (Figure 3B,C). Of the eight serine residues selected as putative ABI1 targets, S48 (Haddock score −155.1 ±8.9 and RMSD* value 4.97 Å) and S85 (Haddock score −160.4 ±12.2 and RMSD* value 7.18 Å) were finally designated as having the highest probability of interaction (Figure 3B,C; Figure S5). In our model, ABI1 residue W300, which is located in a conserved loop, undergoes strong bonding with aromatic or positively charged residues. In the ABI1–ACS7 complex, when ACS7 S48 is oriented toward the ABI1 catalytic pocket, interaction between W300 and Y40 is observed. When ACS7 S48 is targeted to the ABI1 catalytic pocket, ABI1 W300 is close to the F97 residue in ACS7. We suggest, therefore, that the W300 residue in ABI1 may be important for ACS7–ABI1 interaction (Figure 4). This is consistent with previous studies showing that conserved tryptophan residues (ABI1 W300 and HAB1 W385) are important for interaction with ABA-bound ABA receptor and SnRK2.6 [43,46,47,48,49] (Supplementary Figure S6).

### 3.4. ABI1 W300 Is Important for Interaction with ACS7

**Figure 5:**
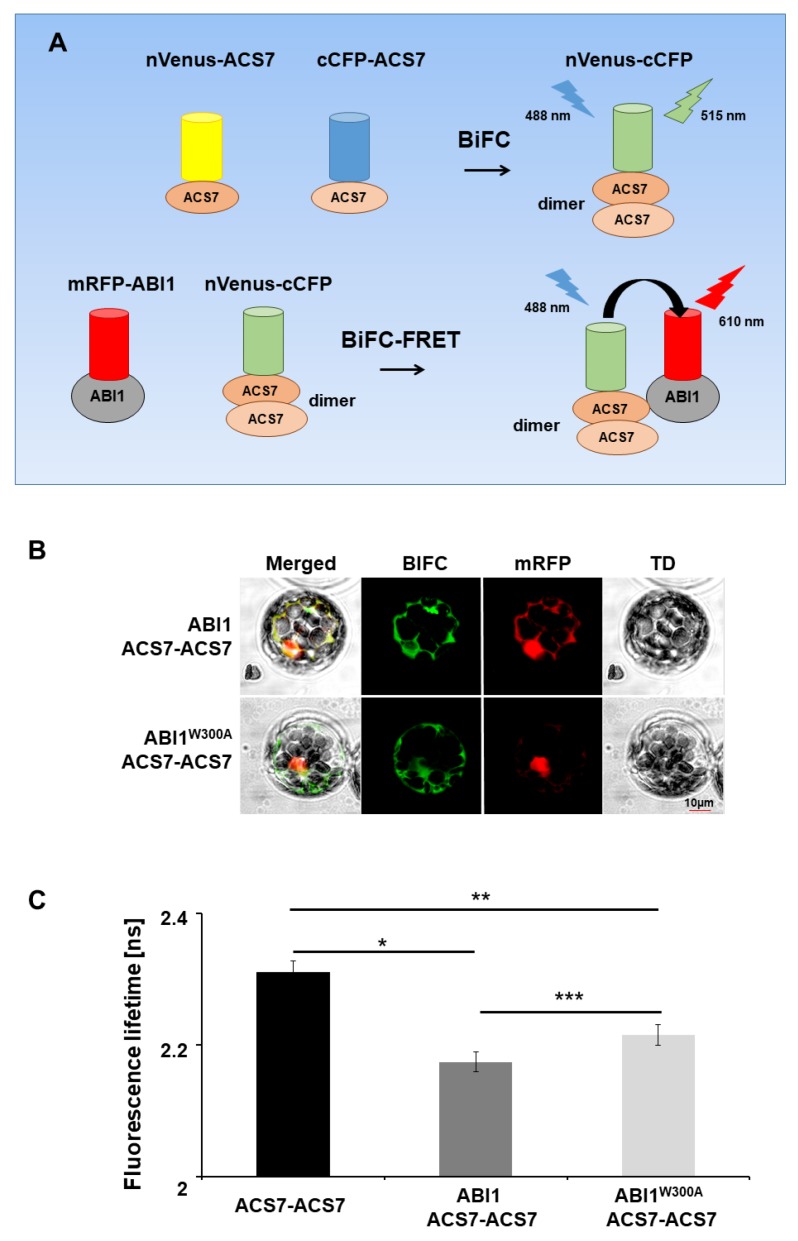
Multicolor BiFC–FRET–FLIM analysis. (**A**) Summary of mcBiFC–FRET–FLIM experiment. Interaction of nVenus–ACS7 and cCFP–ACS7 results in ACS7 dimer reconstruction (nVenus–cCFP) and green light emission (515 nm). Reconstructed ACS7 dimer (nVenus–cCFP donor) excited by 488 nm laser light transfers energy to ABI1–RFP; (**B**) multicolor BiFC–FRET–FLIM analyses of protein interactions between the ACS7 homodimer and WT ABI1 and ABI1 W300A mutant protein in *A. thaliana* protoplasts. (**C**) Co-expression of nVenus–ACS7 and cCFP–ACS7 in protoplasts leads to reconstruction of fluorescent nVenus–cGFP protein by BiFC due to the formation of the ACS7 homodimer. This reconstructed nVenus-cCFP acts as donor. The acceptor mRFP is fused to ABI1 or ABI1 W300A. Fluorescence lifetime of the donor molecule was measured in picoseconds (ps). Error bars indicate the SD (standard deviation, n > 10), and the asterisk indicates a significant difference between the sample in the presence and absence of an acceptor (*p = 0.004; ** *p* = 0.014; *** *p* < 0.00001). Mean value of reconstructed nVenus-cCFP lifetime is *Tamp*: 2.28 ns. χ^2^ ~1 was considered a perfect fit; scale bar, 10 µm.

The ACS7–ABI1 structural model indicates that W300 in the ABI1 sequence is important for the ACS7–ABI1 interaction. To verify this, we used BiFC–FRET–FLIM, a technique that allows in vivo analysis of interactions between more than two partners [50], in our case between ABI1 and the ACS7 homodimer. The ABI2 and HAB1 phosphatases were also included in this analysis. In our experiments, the nVenus–cCFP fluorescence protein, which is reconstituted in the mcBiFC assay, served as FRET donor. Thus, to generate the ACS7 homodimer for BiFC, we tagged ACS7 with the split halves of distinct fluorophores to generate cCFP–ACS7 and nVenus–ACS7. Wild-type and mutated (W300A) forms of ABI1 were tagged with mRFP. Coexpression of ACS7 fusion proteins alone in Arabidopsis protoplasts yielded detectable green fluorescence (Supplementary Figure S6), suggesting that the homodimer had formed efficiently and that the functional nVenus–cCFP fluorophore could be used for FRET experiments. To assess whether the ACS7 homodimer assembles with mutated and non-mutated ABI1, we tested FRET between reconstructed donor nVenus–cCFP molecules (ACS7 homodimer) and mRFP acceptor molecules (ABI1 and ABI1 W300A). The extent of FRET was determined by FLIM as described in the Methods section. When nVenus–ACS7 was co-expressed with cCFP–ACS7 in Arabidopsis protoplasts with ABI1 (or ABI2, or HAB1) and mutated ABI1 W300A, we observed a significant reduction in donor fluorescence lifetime compared with cells transfected with nVenus–ACS7–cCFP–ACS7 alone (*p* = 0.004 and *p* = 0.014; Student’s *t*-test). These results provide further evidence for an interaction between the PP2Cs investigated and ACS7 protein (Figure 5; Figures S2, S7 and S8). However, the fluorescence lifetime of the donor in cells transfected with nVenus–ACS7–cCFP–ACS7 and mRFP–ABI1 W300A constructs was significantly longer than in cells transfected with nVenus–ACS7–cCFP–ACS7 and wild-type mRFP–ABI1 (*p* < 0.00001; Student’s *t*-test), suggesting that mutant ABI1 W300A is impaired in its ability to interact with ACS7 in vivo (Figure 5). The results are consistent with ABI1 W300 being an important amino acid residue for the ACS7–ABI1 interaction.

### 3.5. ABI1 Regulates ACS7 Turnover 

**Figure 6:**
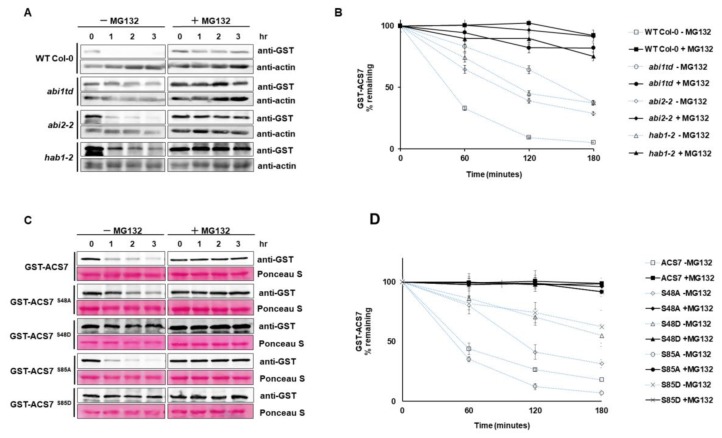
Cell-free degradation assay. (**A**) Protein extracts were prepared from seven-day-old seedlings of WT Col-0, *abi1td*, *abi2* and *hab1-1* mutant plants and then incubated with or without MG132, over the indicated time course. The level of recombinant ACS7 was monitored by immunoblotting, using anti-GST antibody. Actin was used as a loading control and was detected by using anti-actin antibody; (**B**) half-life plot for cell-free degradation of GST–ACS7 in WT Col-0, *abi1td*, *abi2* and *hab1-1* extracts; (**C**) cell-free degradation assay of mutated and nonmutated ACS7 in wild-type protein extracts. The level of recombinant ACS7 was monitored by immunoblotting, using anti-GST antibody. Ponceau staining was used as a loading control; (**D**) half-life plot for cell-free degradation of GST–ACS7, GST–ACS7 S48A, S48D, S85A and S85D mutant. ACS7 protein bands were quantified, using ImageJ software, and normalized, to the control (mock) band (set as 1).

An interaction between ABI1 and ACS7 indicates that ABI1 and possibly other group A PP2Cs, including ABI2 and HAB1, may regulate the stability of ACS7. To directly test their effect on ACS7 stability in a cell-free degradation assay, recombinant GST–ACS7 was incubated with plant extracts from WT Col-0 and the *abi1td, abi2* and *hab1* knockout lines. The protein blots showed the fastest degradation of GST–ACS7 in the WT Col-0 extract without MG132 treatment (Figure 6A). Degradation of recombinant GST–ACS7 protein was significantly delayed in *abi1td* (t_1/2_ < 120 min), *abi2* (t_1/2_ < 120 min) and *hab1* (t_1/2_ < 120 min) extracts without MG132 compared to WT Col-0 (t_1/2_ < 60 min). Further significant delay in GST–ACS7 degradation was observed in all extracts treated with MG132. The stability of GST–ACS7 increased dramatically in *abi1td, abi2, hab1* and WT Col-0 extracts, showing a half-life of up to 180 min (Figure 6B). 

Our modeling approach demonstrated S48 and S85 of ACS7 as potential interaction sites for ABI1 PP2C. To further explore the regulatory relation between ABI1 and ACS7, four different ACS7 mutants were produced that abolish (serine to alanine: S48A and S85A) or mimic phosphorylation of the indicated serine residues (serine to aspartic acid: S48D and S85D). The resulting ACS7 mutants were tested for their role in regulation of ACS7 stability. Recombinant ACS7 S48A, S85A, S48D and S85D mutants were incubated with plant extracts from WT Col-0 with and without MG132 (Figure 6C,D). Degradation of ACS7 S48A, S48D and S85D mutants was significantly delayed in plant extracts without MG132, as compared to wild-type ACS7 without MG132 treatment. The half-life of ACS7 containing S48A mutation was less than 120 min (Figure 6C,D), while the t_1/2_ of ACS7 containing S48D or S85D mutations was significantly longer than 180 min (t_1/2_ > 180 min). Interestingly, without MG132 treatment, the ACS7 S85A mutant degraded even faster than the wild-type protein (t_1/2_ < 60 min). The half-life of mutated and wild-type ACS7 in plant extracts treated with MG132 was also much longer than 180 min (Figure 6C,D). These results demonstrate that S48 and S85 residues of ACS7, recognized as potential targets of ABI1 PP2C, determine the stability of ACS7. However, only S48 stabilizes ACS7, even in its non-phosphorylated form.

**Figure 7:**
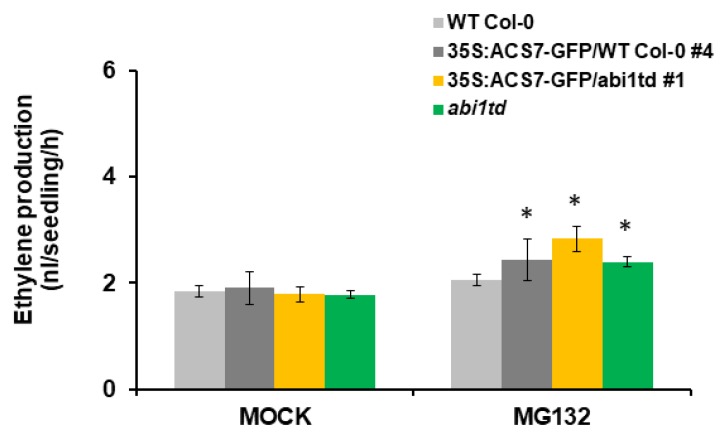
MG132 increases ethylene production in transgenic plants expressing 35S:ACS7–GFP vector construct. Two-week old seedlings were treated with MG132 (or equivalent control) in GC vials, and ethylene in the headspace was measured. Experiments were performed in triplicate, with consistent results (n = 5–10 replicates for each treatment). Error bars show standard deviation. Asterisks indicate a statistically significant difference at *p* < 0.05.

To further test whether ABI1 PP2C regulates ACS7 stability, we generated transgenic plants expressing 35S:ACS7–GFP in *abi1td* and WT Col-0 backgrounds. Treatment with proteasome inhibitor MG132 increased ethylene levels in transgenic plants, compared to controls in both *abi1td* and WT Col-0 plants (Figure 7). In addition, ethylene biosynthesis due to ACS7 overexpression was significantly higher in the *abi1td* background (Figure 7), suggesting that ABI1 PP2C plays an important regulatory role in ACS7 stability. Overall, our results demonstrate that group A PP2Cs’ members are key factors affecting ACS7 turnover.

## 4. Discussion

In this work, we provide evidence that ABI1 and ABI1-like PP2Cs are involved in the regulation of ethylene biosynthesis via interaction with ACS7. Multicolor BiFC and pull-down assays demonstrated that ACS7 interacts with the PP2Cs ABI1, ABI2 and HAB1 (Figure 2), while mcBiFC–FRET–FLIM analysis showed that the ACS7 homodimer assembles with the PP2Cs, predominantly in the cytoplasm (Figure 5; Figure S7). Computational modeling and subsequent experimental verification demonstrated that the tryptophan at position 300 in the ABI1 amino acid sequence is crucial for interaction with ACS7 (Figure 3 and Figure 5). Furthermore, we showed that ACS7 stability is significantly increased in total protein extracts isolated from *abi1td*, *abi2* and *hab1-1* knockout lines (Figure 6A,B), suggesting that ABI1, ABI2 and HAB1 PP2Cs regulate turnover of the ACS7 protein in Arabidopsis. Consistent with this, we observed increased ethylene production in ACS7-overexpressing plants in the *abi1td* background compared to overexpression in the WT Col-0 background (Figure 7). In addition, we found that serine residues 48 and 85, potential interaction sites for ABI1 PP2C, regulate ACS7 protein stability (Figure 6C,D).

The results presented here advance our understanding of ACS7:group A PP2C interactions in several important ways. Among the more interesting features of ACS7 is its truncated C-terminal domain [18,23,51], suggesting that sophisticated mechanisms control ACS7 action. However, the regulatory mechanisms coordinating ACS7 function seem to be, in principle, similar to those observed for type I and type II ACSs [14,25,52,53,54,55]. Reversible phosphorylation plays a major role in the regulation of ACS7 [12,19,44], although, to date, only a single protein kinase has been shown to interact with ACS7: CDPK16 catalyzes multiple phosphorylation events that determine ACS7 catalytic activity [19]. Our finding that ACS7 interacts with the PP2Cs ABI1, ABI2 and HAB1 (Figure 2 and Figure 5; Figure S5) suggests that dephosphorylation is likewise important for ACS7 function. ABI1 was previously shown to dephosphorylate various protein kinases, including MAP kinases [25,31,56,57] and the SnRK1 and SnRK2 kinases [58,59,60,61,62]. ABI1 was also found to dephosphorylate the MAPK site in type I ACS [25]. Dephosphorylation by ABI1 causes the inactivation of its substrates, in line with its function as a negative regulator in ABA signaling [25,59,60,61,62]. However, despite this important insight into ABI function, the consensus amino acid sequence for substrate recognition by ABI1 (or related PP2Cs) had not previously been defined. Therefore, we asked, what is the molecular mechanism of ACS7–ABI1 interaction? To answer this question, we used multiple approaches, looking for both the domains and the critical residues in both proteins that are necessary for complex assembly. According to the InterPro database, structurally, the ACS7 protein forms a single domain, known as pyridoxal phosphate-dependent transferase, which in turn comprises two subdomains. There are also unstructured fragments at both the N-terminus and C-terminus (www.ebi.ac.uk/interpro/protein/Q9STR4). This assessment of the ACS7 domain structure is largely consistent with our modeling results and suggests that interaction between ABI1 and ACS7 has a structural basis. Intriguingly, however, we found that all the N- and C-terminal ACS7 deletion mutants we generated (Figure 2) were capable of interacting with ABI1. This indicates that there are multiple ABI1 docking sites on the ACS7 protein. Significantly, in this regard, serine residues S48 and S85 are located within the Δ1 and Δ2 ACS7 deletion regions, respectively, while S272 is located within the Δ3 and Δ4 ACS7 deletion fragments. This is perhaps consistent with the ABI1–ACS7 interaction being subject to spatial and temporal regulation. On the other hand, we cannot exclude the possibility that ACS7 mutant proteins, especially small deletion fragments, adopt different conformations that affect the specificity or even spectrum of interacting proteins. 

We first attempted to identify key residues within ABI1 that are important for ACS7–ABI1 interaction, using a modeling approach (Figure 3 and Figure 4; Figures S2 and S3). Our model implied that W300 was essential for ABI1 binding to ACS7. The functional consequence of mutation at the W300 residue was that the interaction of ABI1 with ACS7 was attenuated but not abolished, suggesting that ABI1–ACS7 assembly involves other residues. Interestingly, the same residue has been noted to be involved in PYL–ABI1 complex formation [47]. Clade A PP2Cs interact with PYR/PYL proteins via a small recognition loop that contains a conserved tryptophan residue, which is W300 in ABI1 [47,59,63]. This W300 residue, of which the indole group has been called the “lock” [46], is crucial for binding the closed gate and latch loops of the receptor. Based on our modeling results and the above findings, we propose that this “lock” site stabilizes the ABI1–ACS7 complex, where the conserved tryptophan residue in ABI1 (W300) forms a water-mediated contact with ACS7, making it critical for interaction with the gate and latch loops [46,47,64,65]. These data demonstrate that the interaction interface between ABI1 and ACS7 overlaps with the PYL–PP2C interface, lending further support to a conserved mechanism of interaction between PP2Cs and their targets.

The functional consequences of ACS7–ABI1 interaction are predictable, since phosphorylation/dephosphorylation events are required for the normal function of ACSs [54,55]. Cyclic phosphorylation/dephosphorylation events are thought to promote ubiquitination and ACS protein degradation. In addition to the involvement of several known protein kinases in ACS turnover [11,55,66], protein phosphatases have also been shown to regulate ACS abundance [25,54]. Skotkke and coworkers demonstrated that protein phosphatase 2A (PP2A) activity has the opposite effect on turnover of type I and II ACC synthases [54]. Thus, while PP2A-dependent dephosphorylation immediately reduces ACS6 (type I) protein levels, PP2A dephosphorylation of ACS5 leads to accumulation of this type II synthase. Post-translational regulation of the ACS gene family was highlighted by the identification of ubiquitin–proteasome system-dependent control of ethylene production [44,53,54,67,68,69,70]. Members of the PP2C family were also implicated in the regulation of ACS abundance. For instance, ABI1 and ABI2 target ACS6 for degradation [25]. Although ABI1 and ABI2 are functionally similar, they can have different interaction partners. Thus, ABI1, but not ABI2, interacts with MAPKKK18 [31]; similarly, only ABI1, and not ABI2, is able to interact with transcription factor ATHB6 from the ABA response pathway [71]. It seems that, under some conditions at least, PP2Cs may act with high specificity regarding substrates, while operating redundantly in other situations.

Despite recent advances in understanding the mechanism of type I and type II ACS degradation, the factors that regulate ACS7 protein turnover still remain unclear. Identification of ABI1, ABI2 and HAB1 as key proteins regulating ACS7 stability shows that ACS dephosphorylation is accomplished by particular group A PP2C members. Why is the regulation of ACS7 stability so complex? One of the reasons may be connected to development, with different PP2Cs taking the lead in ACS7 turnover, at different developmental stages or in different organs. The triple mutant *abi1 abi2 hab1* is hypersensitive to ABA and accumulates more ethylene than WT [72]. Furthermore, Luo and co-workers found that ACS7 transcript levels in the *abi1 abi2 hab1* triple mutant after ABA treatment were elevated at least two times in comparison to similarly treated WT plants and were three times higher than in the *abi1-1* mutant [72]. This clearly points to the fact that ACS7 stability is under the control of ABI1, ABI2 and HAB1. 

In a cell-free degradation assay, the half-life of N-tagged GST–ACS7 in a WT-derived protein extract is about 30 min. However, incubation of GST–ACS7 with a protein extract prepared from PP2C-knockout plants markedly extends its half-life. The half-life of recombinant ACS7 was previously reported by Lyzenga et al., at about 15 min, and by Lee et al., at around 30 min [14,42]. This means that our results are consistent with the literature and that the delay in ACS7 degradation in protein extracts from PP2C-knockout plants is significant. Strikingly, this difference is not seen when the cell-free experiments are conducted in dark conditions, which is in agreement with previous reports that ACC synthases are stable in the dark and are only degraded when illuminated [18,20,21]. These findings accord with the well-known light-dependence of ethylene biosynthesis in plants [73,74,75]. 

**Figure 8:**
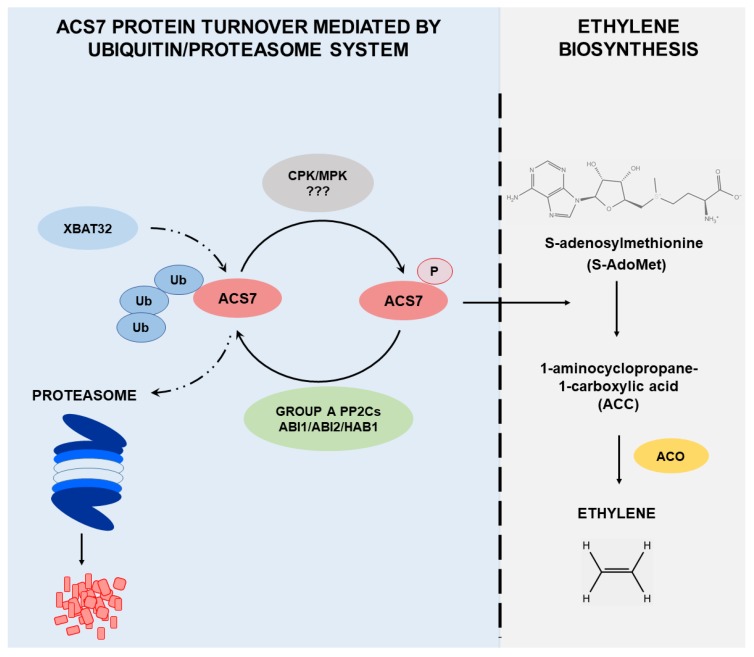
Overview of the regulation of ACS7 protein turnover. ACS7 is the only type III ACC synthase in Arabidopsis and is characterized by a unique structure with no regulatory domains, despite the fact that ACS7 protein undergoes proteasomal degradation. Phosphorylation mediated by CDPK and/or MAP kinases results in ACS7 activation and an increase in ethylene biosynthesis. Protein phosphatases type A (ABI1, ABI2 and HAB1) remove the phosphate group from ACS7, which in turn inhibits its activity and promotes ubiquitination mediated by the XBAT32 E3 ubiquitin ligase. After ubiquitination, ACS7 is immediately directed for degradation via the proteasome pathway, and ethylene biosynthesis driven by ACS7 is thus restricted.

Taken together, these data reveal complex mechanisms of regulation of ACS7 (Figure 8), which has a unique structure and function among the ACC synthases involved in ethylene biosynthesis. Compared to type I ACSs, the relative lack of specificity in ACS7 dephosphorylation by group A PP2Cs represents an intriguing mechanism for the regulation of protein stability and abundance. The unique role of ABI1 in regulating the stability of type I and type II ACSs [42] also suggests that ACS7 requires precise temporal or dose-dependent regulation. Furthermore, light is involved in the control of ACS7 stability in a way that is not yet fully elucidated. It seems likely that these various modes of regulation of ACS7 activity and stability involve multiple interacting posttranslational modifications. Therefore, to fully understand the complex regulation of ACS7 activity and turnover, it will be necessary to determine the interplay between phosphorylation, ubiquitination and SUMOylation. Future identification of posttranslational modification sites and/or light response elements will be necessary for a detailed understanding of ACS7 regulation.

## Supplementary Materials

The following are available online at https://www.mdpi.com/2073-4409/9/4/978/s1. Figure S1: Subcellular localization of the ACS7 protein in the Arabidopsis protoplasts. Figure S2: Multicolor BiFC–FRET–FLIM controls. Figure S3: BiFC controls. Figure S4: Testing the proposed macromolecular docking model. Figure S5: Summary of Haddock predictions. Figure S6: Structural insights into the interaction mechanisms in the PYL1/SnRK2.6–ABI1 and ACS7–ABI1 protein complexes. Figure S7: Multicolor BiFC–FRET–FLIM. Figure S8: Emission spectrum of nVenus-cCFP BiFC in protoplasts. Table S1: List of primers used in this study. Table S2: Evaluation of ACS7–ACS7 (homodimer) structures from macromolecular docking with calculated potential energy in OPLS3 force field. Table S3: Evaluation of ACS7–ABI1 docking structures from macromolecular docking with calculated root-mean-square deviation (*RMSD*).
