# Supplementary material for: Protein Phosphatases Type 2C Group A Interact with and Regulate the Stability of ACC Synthase 7 in Arabidopsis
Source: Cells. 2020 Apr 15;9(4):978. doi: 10.3390/cells9040978 (PMC7227406; doi:10.3390/cells9040978)
Supplement: Supplementary file 1 [file cells-09-00978-s001.pdf]

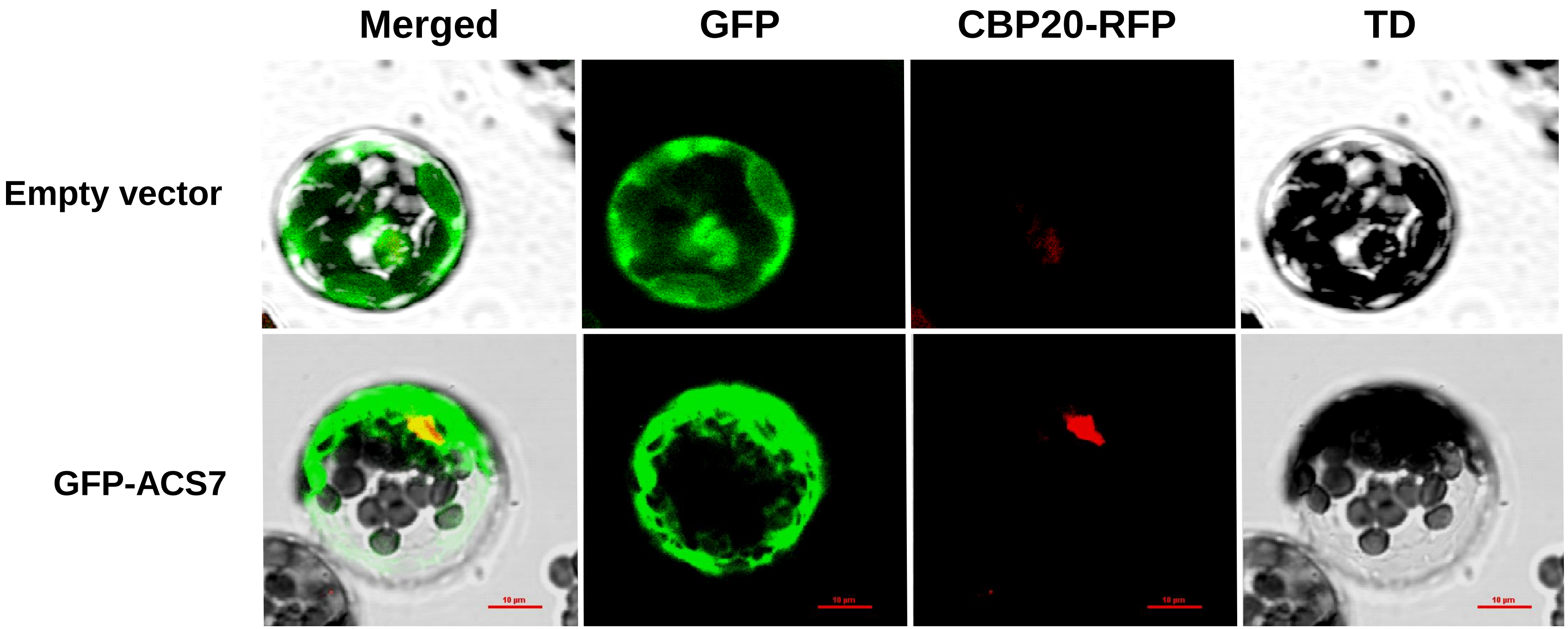

Supplementary Figure S1. Subcellular localization of the ACS7 protein in the *Arabidopsis* protoplasts.
Microscopy images show nuclear localization of the GFP-ACS7 fusions in protoplasts isolated from leaves of Arabidopsis wild type plants. CBP20-RFP was used as a marker of nuclear localization. Empty pSITE-2CA was used as negative control. Scale bar, 10 µm.

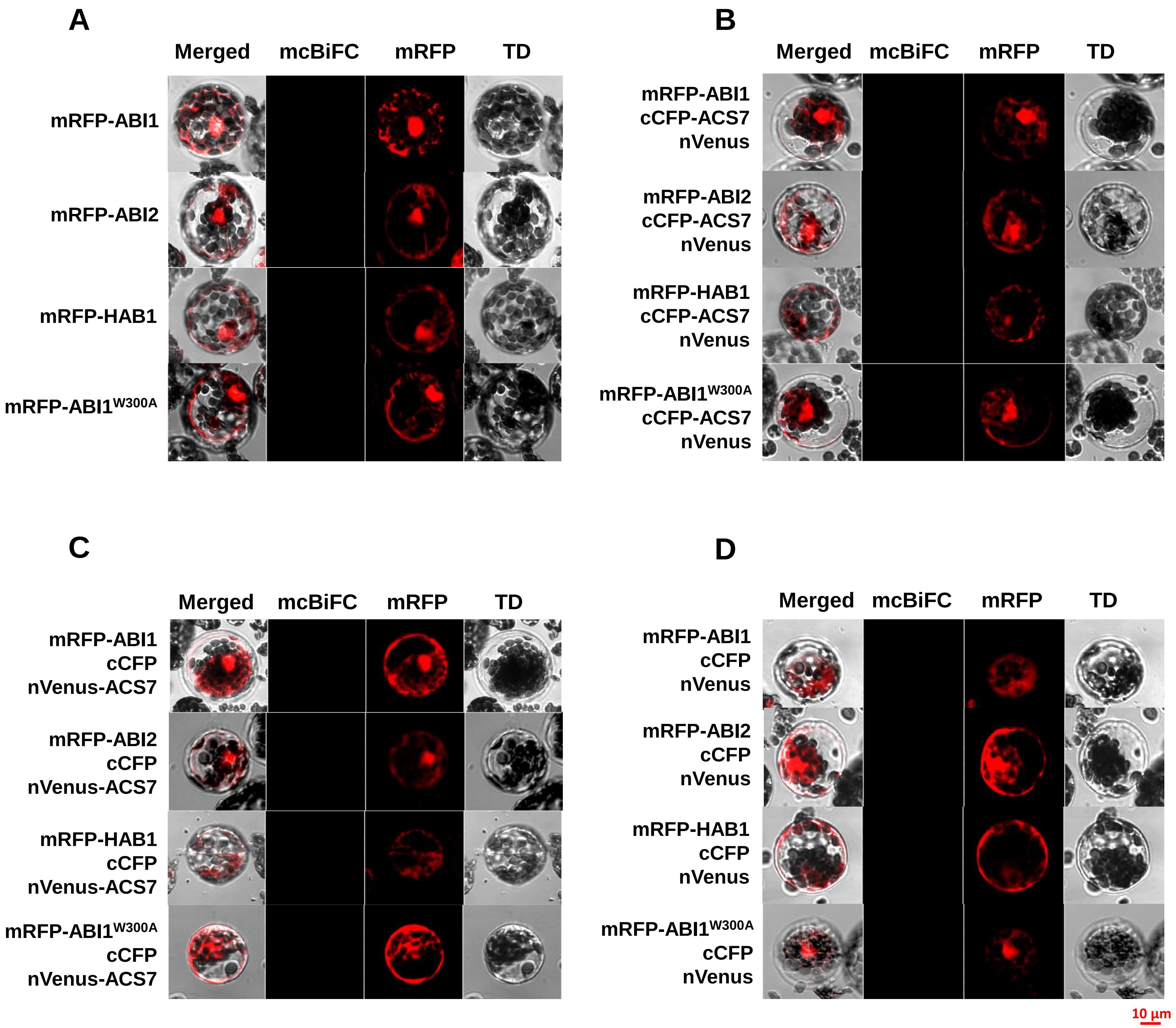

Supplementary Figure S2. Multicolor BiFC-FRET-FLIM controls.
Control transformations confirming the specificity of interaction between the ACS7 homodimer and PP2Cs. **A.** Protoplasts transformed with ABI1/ABI2/HAB1 and ABI1W300A fused with mRFP (mRFP-PP2C); **B.** Arabidopsis protoplasts transformed with mRFP-PP2C, cCFP-ACS7 and empty vector with nVenus; **C.** Arabidopsis protoplasts transformed with PP2C-mRFP, ACS7-nVenus and empty vector with cCFP **D.** Co-expression of mRFP-PP2C with cCFP and nVenus empty vectors. As expected no signal is observed in the donor (mcBiFC) channels. Scale bar, 10 µm.

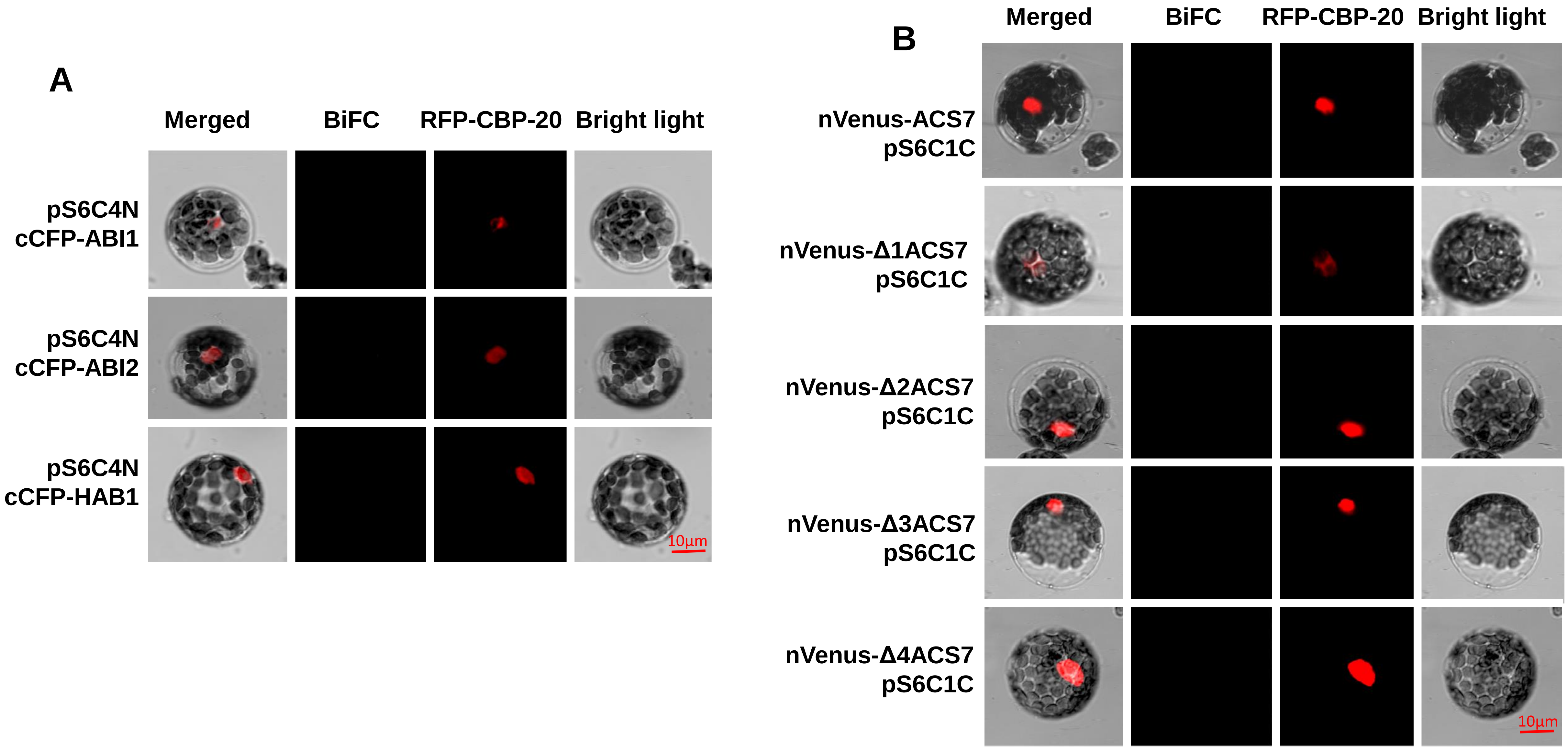

Supplementary Figure S3. BiFC controls.

Control transformations confirming the specificity of interaction between the ACS7/Δ1/Δ2/Δ3 /Δ4ACS7 and PP2Cs (ABI1/ABI2/HAB1). **A**. Protoplasts transformed with ABI1/ABI2/HAB1 fused with cCFP (cCFP-PP2C) and empty vector with nVenus (pS6C4N). **B.** Arabidopsis protoplasts transformed with nVenus- ACS7/Δ1/Δ2/Δ3 /Δ4ACS7 and empty vector with cCFP (pS6C1C). The signal is not observed in the GFP channels. Scale bar, 10 μm.

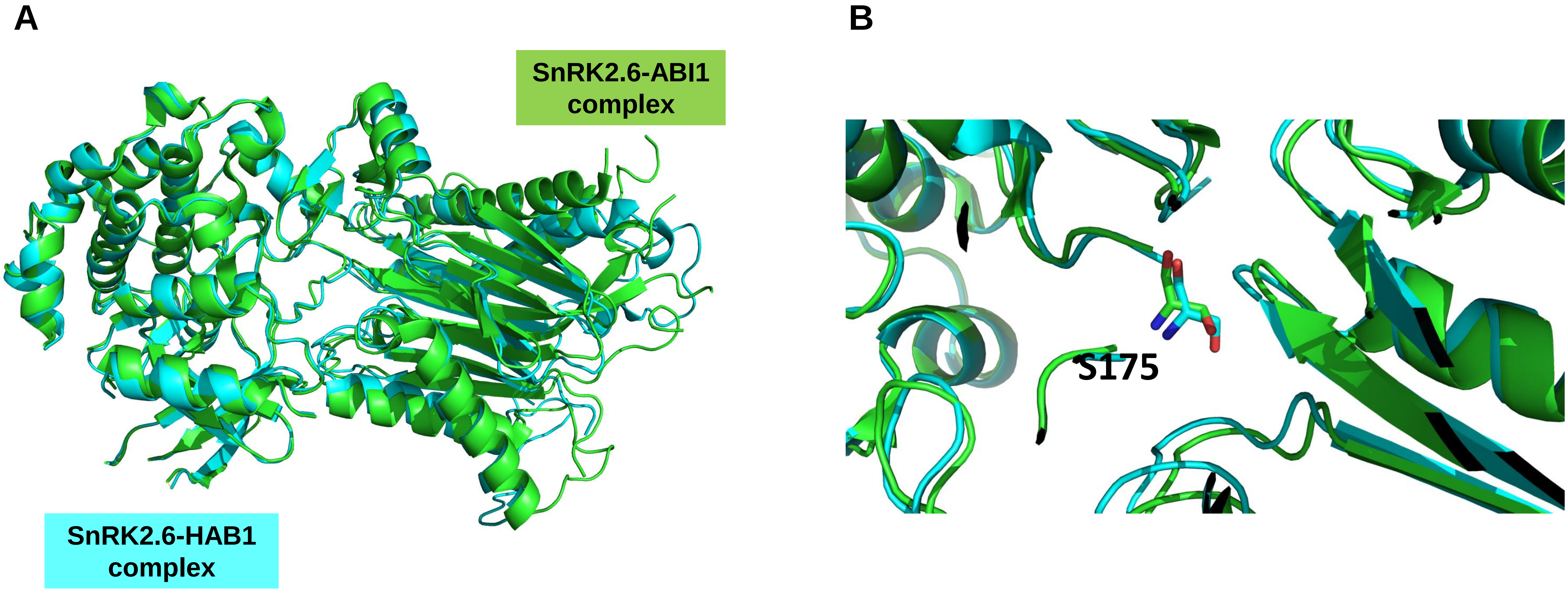

Supplementary Figure S4. Testing the proposed macromolecular docking model.
A. Superposition of the SnRK2.6-HAB1 complex (PDB code: 3UJG) and the SnRK2.6-ABI1 complex – the structural model represents the best result from *in silico* macromolecular docking experiments using Haddock software**; B.** Close up view of the aligned protein complex with serine residue (S175) labelled and highlighted as sticks. Root-mean-square-deviation (RMSD) value for the position of the serine residue (S175) equals 0.9 Å**.**

A

Target	Haddock Score (HS)	Rank	RMSD*
ACS7^{S24}	-151.8 +/- 1.7	3	15.825
**ACS7^{S48}**	**-155.1 +/- 8.9**	**2**	**4.979**
ACS7^{S52}	-142.9 +/- 1.4	4	13.324
**ACS7^{S85}**	**-160.4 +/- 12.2**	**1**	**8.673**
ACS7^{S182}	-134.9 +/- 7.9	6	9.170
ACS7^{S183}	-130.5 +/- 25.6	7	6.877
ACS7^{S272}	-118.8 +/- 4.1	8	9.090
ACS7^{S413}	-138.2 +/- 1.4	5	16.107

B

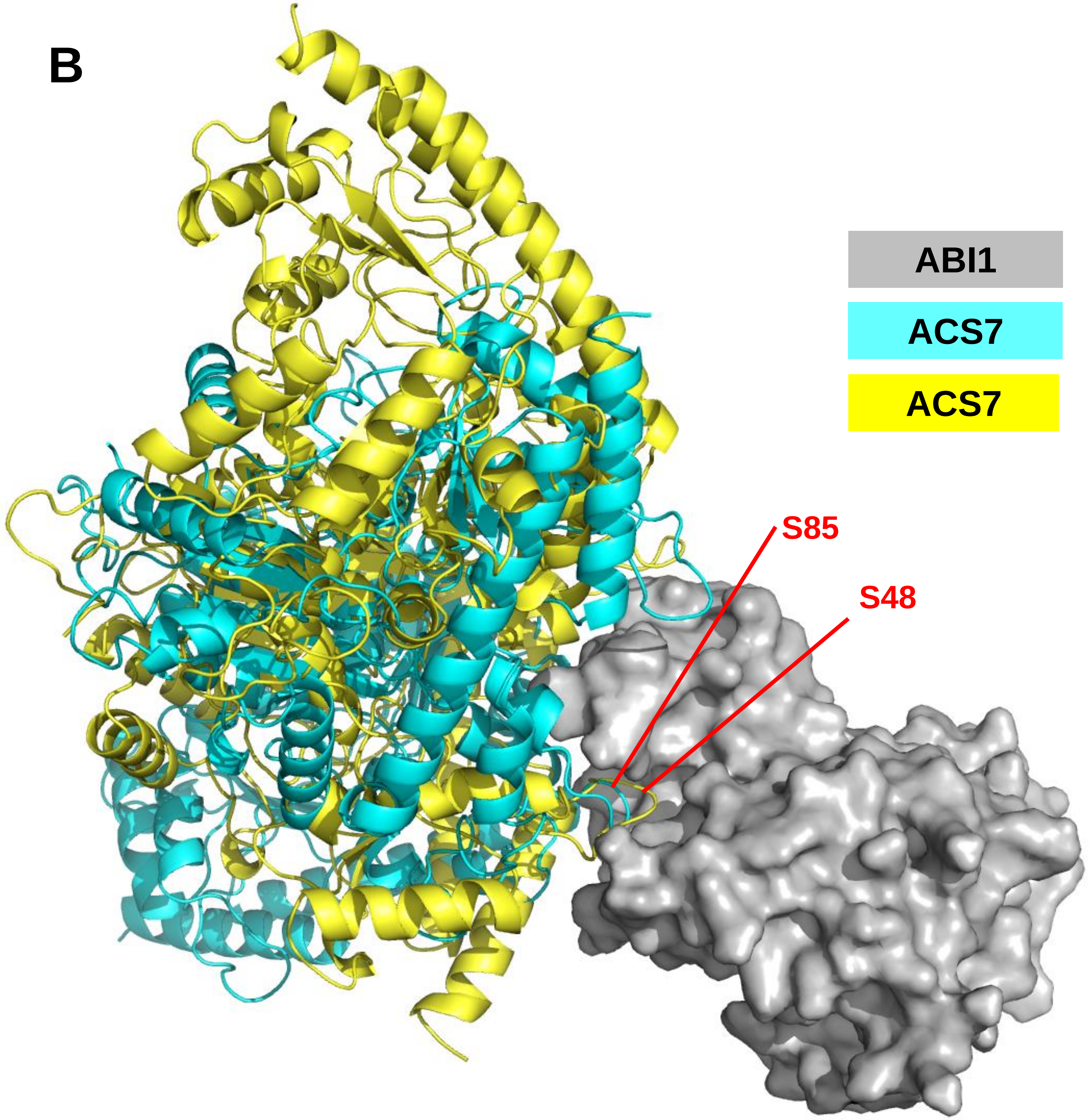

Supplementary Figure S5. Summary of Haddock predictions.

A. Target serine residues in the ACS7 protein were used as constraints to build the complex with ABI1 and docked into the proposed binding site of the ABI1 protein; thermodynamic data of the investigated complexes were calculated using the Haddock scoring function. Rank refers to the ranking of that particular ABI1-ACS7 complex by the Haddock scoring function. **B**. The top-ranked ABI1-ACS7 complexes according to the Haddock scoring function and visual analysis. ACS7 protein is shown in cartoon representation with the target serine residues indicated; ABI1 protein is shown as a grey surface.

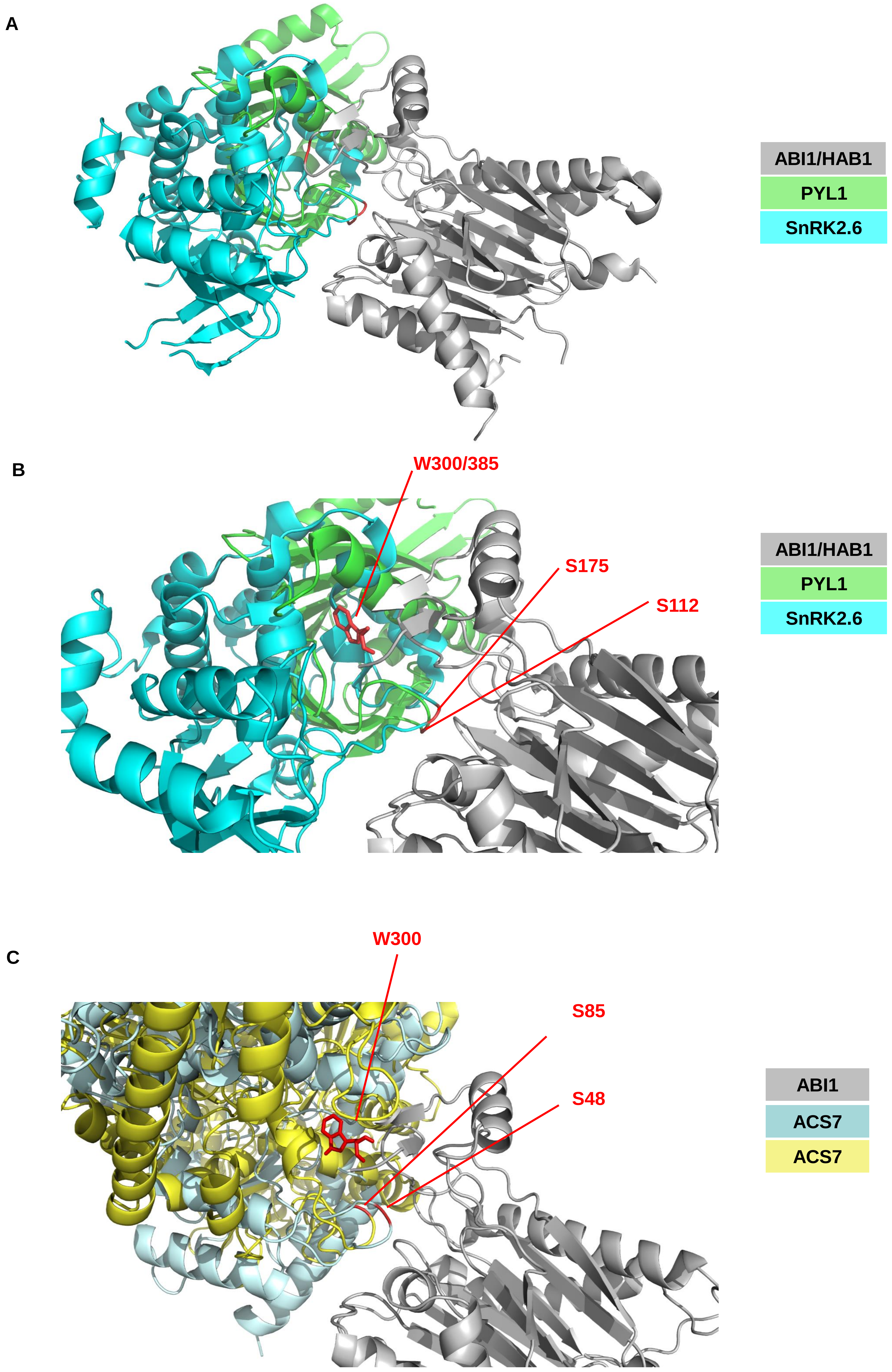

Supplementary Figure S6. Structural insights into the interaction mechanisms in the PYL1/SnRK2.6-ABI1 and ACS7-ABI1 protein complexes.

A. Ribbon diagram of the aligned SnRK2.6-HAB1/ABI1-PYL1 complex: ABI1 and HAB1 are in grey; SnRK2.6 is shown as a blue cartoon and PYL1 is shown in green; **B.** In the structure, residues that take part in the protein-protein interaction are highlighted (S residues of PYL1 and SnRK2.6 proteins; the W300 residue of ABI1 is highlighted as red sticks); **C.** Superposition of ACS7 structures and ABI1, with residues involved in the interaction highlighted. ACS7 in different orientations is shown as green, yellow and cyan cartoons. ABI1 is shown in grey.

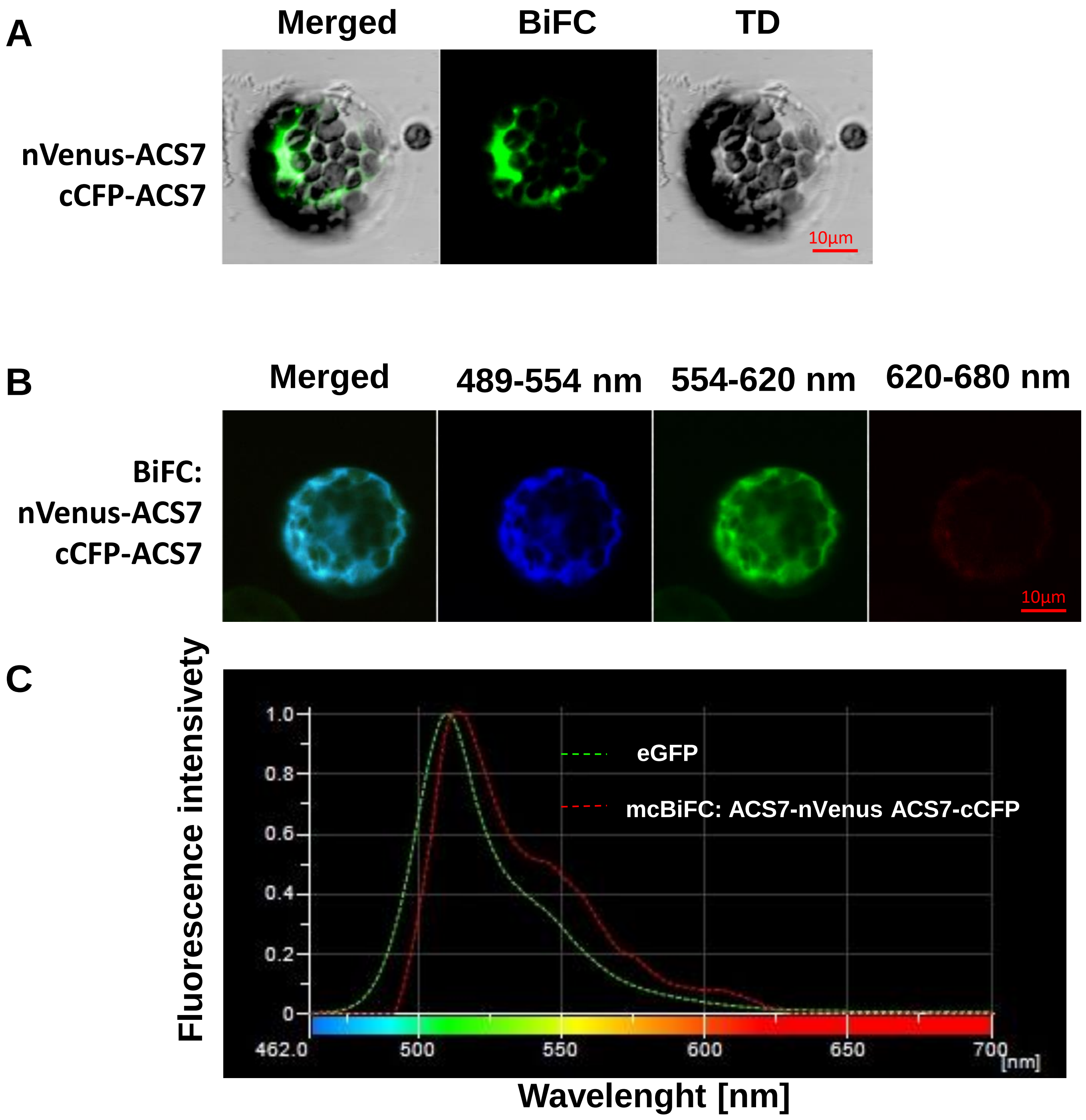

Supplementary Figure S7. Multicolor BiFC-FRET-FLIM.

A. Multicolor BiFC-FRET-FLIM analyses of protein interactions between the ACS7 homodimer and PP2C proteins (ABI1, ABI2, HAB1, ABI1 W300A) in *A. thaliana* protoplasts. Co-expression of nVenus-ACS7 and cCFP-ACS7 in protoplasts leads to reconstruction of a fluorescent nVenus-cCFP protein by mcBiFC due to the formation of the ACS7 homodimer. This reconstructed nVenus-cCFP acts as donor. The acceptor mRFP is fused to ABI1, ABI1 W300A, ABI2 or HAB1. **B.** Fluorescence lifetime of the donor molecule was measured in picoseconds [ps]. Error bars indicate the SD (standard deviation, n > 10), and the asterisk indicates a significant difference between the sample in the presence and absence of an acceptor (* $p = 0.004$, $p = 0.014$, $p = 0.006$, $p = 0.005$; ** $p < 0.00001$, respectively). Mean value of reconstructed nVenus-cCFP lifetime is *Tamp*: 2.28 ns. χ^2 ~1 was considered a perfect fit. Scale bar, 10 µm. There is no significant difference in interaction with ACS7 among the investigated PP2Cs.

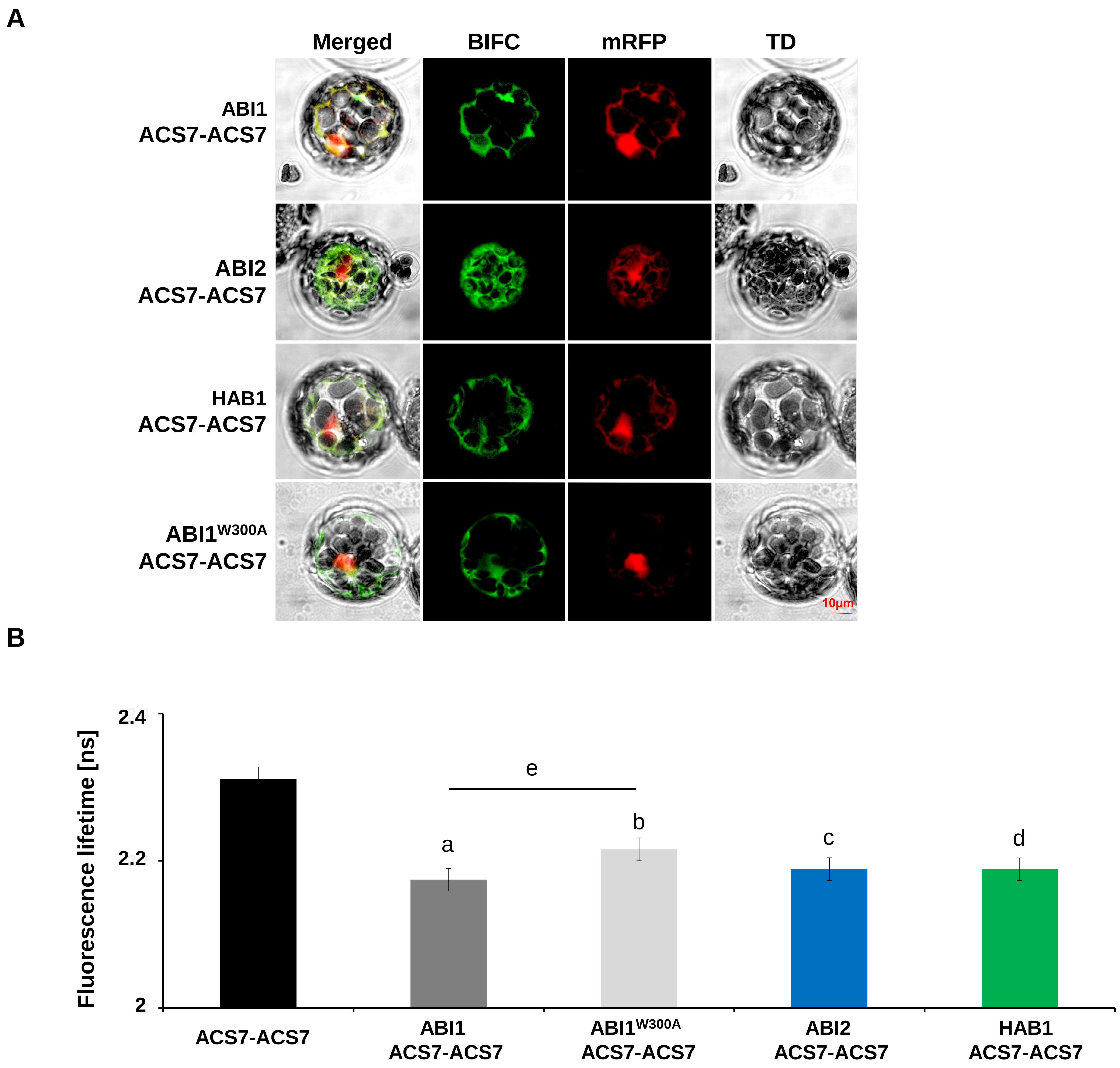

Supplementary Figure S8. Emission spectrum of nVenus-cCFP BiFC in protoplasts.
A. Visualization of nVenus-ACS7 and cCFP-ACS7 BiFC with 488 nm laser, standard confocal detector and fluorescence emission filter set (500–550 nm); **B.** After nVenus-ACS7 and cCFP-ACS7 complex were transiently expressed in protoplasts, the emission spectrum of the reconstructed fluorophore was measured by Nikon A1R confocal microscope with a 488 nm laser and spectral detector in range 489-680 nm with 6 nm resolution, according to the instruction manual of Nikon Instruments; **C.** Comparison of eGFP fluorescence emission spectrum and nVenus-cCFP emission spectrum visualized in Nikon NIS-Elements software.

Supplementary Table S1. List of primers used in this study.

List of primer sequences used for vector construction and mutagenesis.

**Name of primers**	**Primer sequences 5’->3’**
ABI1 Reverse	TCAGTT CAAGGG TTTGCT CTTGAG TTTCC
ABI1 Forward	ATGGAG GAAGTA TCTCCG GCGATC
ABI2 Reverse	TCAATT CAAGGA TTTGCT CTTGAA TTTCC
ABI2 Forward	ATGGAC GAAGTT TCTCCT GCAGTC G
HAB1 Reverse	CATTCA GGTTCT GGTCTT GAACTT TCTTTG
HAB1 Forward	CACCAT GATGGA GGAGAT GACTC
AHG1Reverse	TTACTG AGAGCT ATTCTT GAGATC AATGAC
AHG1Forward	CACCAT GACTGA AATCTA CAGAAC AATTTC
ABI1 W300A Reverse	CGAGCT CCATTC GCCTGA ATCACT TTC
ABI1 W300A Forward	GAAAGT GATTCA GGCGAA TGGAGC TCG
ACS7 S48A Reverse	GAAGGG TTATGC GCTTCG TCGTAA G
ACS7 S48A Forward	CTTACG ACGAAG CGCATA ACCCTT C
ACS7 S48D Reverse	GAAGGG TTATGA TCTTCG TCGTAA G
ACS7 S48DForward	CTTACG ACGAAG ATCATA ACCCTT C
ACS7 S85A Reverse	AGCTCC TTTCGC TCCCCA CATC
ACS7 S85A Forward	GATGTG GGGAGC GAAAGG AGCT
ACS7 S85D Reverse	GGAGCT CCTTTA TCTCCC CACATC
ACS7 S85D Forward	GATGTG GGGAGA TAAAGG AGCTCC
ACS7 Reverse C-tag	AAACCT CCTTCG TCGGTC CA
ACS7 Forward C-tag	CACCAT GGGTCT TCCTCT AA
ACS7 Reverse N-tag	TCAAAA CCTCCT TCGTCG GTC
ACS7 Forward N-tag	CACCGG TCTTCC TCTAAT GAT
Delta1 ACS7 Reverse	TCAGTA GTCTTG AAACAA TGCGTT
Delta2 ACS7 Reverse	TCAGAC ACCGGA AGGGTT AT
Delta4 ACS7 Forward	CACCAT GCACGG TCTCAA AACT
Delta6 ACS7 Forward	CACCCG TGACGC GAACAT TAGAGT

Supplementary Table S2. Evaluation of ACS7-ACS7 (homodimer) structures from macromolecular docking with calculated potential energy in OPLS3 force field.
Summary table of evaluated conformers of ACS7-ACS7 homodimers from macromolecular docking simulation with potential energy after geometry optimization.

ACS7 dimer name	E_{pot} (kcal*mol^{-1})	Forcefield
**ACS7_dimer_1**	-38681,9	OPLS3
**ACS7_dimer_2**	-38884,2	OPLS3
**ACS7_dimer_3**	-38937	OPLS3
**ACS7_dimer_4**	-39183,2	OPLS3
**ACS7_dimer_5**	-39123,6	OPLS3
**ACS7_dimer_6**	-38993,2	OPLS3
**ACS7_dimer_7**	-38978	OPLS3
**ACS7_dimer_8**	-37100,4	OPLS3
**ACS7_dimer_9**	-39086,9	OPLS3
**ACS7_dimer_10**	-38901,4	OPLS3
**ACS7_dimer_11**	-39031,3	OPLS3
**ACS7_dimer_12**	-38997	OPLS3
**ACS7_dimer_13**	-38943,8	OPLS3
**ACS7_dimer_14**	-38980,3	OPLS3
**ACS7_dimer_15**	-38960,3	OPLS3
**ACS7_dimer_16**	-39046,4	OPLS3
**ACS7_dimer_17**	-38888,2	OPLS3
**ACS7_dimer_18**	-38987,9	OPLS3
**ACS7_dimer_19**	-38797,7	OPLS3
**ACS7_dimer_20**	-38879,9	OPLS3
**ACS7_dimer_21**	-38994,3	OPLS3
**ACS7_dimer_22**	-38831,5	OPLS3
**ACS7_dimer_23**	-38949,1	OPLS3
**ACS7_dimer_24**	-38893,9	OPLS3
**ACS7_dimer_25**	-39052,4	OPLS3
**ACS7_dimer_26**	-38885,3	OPLS3
**ACS7_dimer_27**	-38918,2	OPLS3
**ACS7_dimer_28**	-39002,3	OPLS3
**ACS7_dimer_29**	-38820,1	OPLS3

Supplementary Table S3. Evaluation of ACS7-ABI1 docking structures from macromolecular docking with calculated root-mean-square deviation (*RMSD*).

Summary table of evaluated ACS7-ABI1 model structure complexes depending on the selected serine residue as target with Haddock score and RMSD value. (*) RMSD calculated between Ca atoms of the targeted serine of the ACS7-ABI1 complex and serine 175 of the kinase substrate (SnRK2.6)-ABI1 complex. Both complex structures were aligned.

ACS^{S24}- ABI1	Haddock score	RMSD*	E_{vdw}	E_{elec}	E_{desol}
Cluster1	-151.8 +/- 1.7	15.825	-57.9 +/- 3.1	-236.7 +/- 7.3	-53.8 +/- 6.2
cluster7	-133.2 +/- 13.8	22.097	-45.2 +/- 5.2	-277.6 +/- 24.8	-36.0 +/- 13.9
cluster6	-118.5 +/- 12.9	15.978	-38.6 +/- 6.2	-107.7 +/- 17.8	-68.1 +/- 10.7
cluster8	-114.9 +/- 8.9	16.446	-48.2 +/- 6.1	-190.1 +/- 38.4	-34.8 +/- 3.0
cluster2	-114.7 +/- 5.4	19.917	-37.8 +/- 2.1	-139.4 +/- 30.2	-55.7 +/- 6.0
cluster4	-111.6 +/- 11.5	18.017	-52.0 +/- 3.8	-133.4 +/- 32.4	-42.2 +/- 8.4
cluster5	-108.6 +/- 12.3	19.529	-56.4 +/- 6.3	-135.7 +/- 43.9	-27.0 +/- 8.6
cluster3	-105.4 +/- 5.1	22.327	36.9 +/- 6.0	-161.9 +/- 25.0	-38.7 +/- 6.0
cluster9	-101.6 +/- 14.6	15.003	-34.6 +/- 1.8	-162.2 +/- 42.9	-39.9 +/- 8.8
**ACS^{S48}- ABI1**	**Haddock score**	**RMSD***	E_{vdw}	E_{elec}	E_{desol}
cluster1	-155.1 +/- 8.9	4.979	-49.5 +/- 12.6	-408.5 +/- 73.	-24.1 +/- 13.1
cluster2	-135.9 +/- 2.8	4.355	-54.2 +/- 9.3	-152.0 +/- 19.6	-51.5 +/- 4.2
cluster5	-134.9 +/- 4.0	7.346	-30.4 +/- 7.1	-344.8 +/- 28.2	-36.7 +/- 3.5
cluster3	-124.2 +/- 1.7	9.116	-52.8 +/- 5.2	-176.8 +/- 48.7	-36.2 +/- 10.4
cluster7	-110.0 +/-9.3	6.274	-45.3 +/- 4.6	-123.8 +/- 35.3	-40.0 +/- 7.5
cluster8	-109.8 +/- 21.0	10.547	-36.5 +/- 5.2	-147.8 +/- 45.7	-43.9 +/- 13.7
cluster6	-100.8 +/- 2.4	16.513	-45.2 +/- 3.9	-58.0 +/- 13.3	-49.0 +/- 3.7
cluster4	-95.8 +/- 1.2	5.792	-31.6 +/- 3.6	-228.9 +/- 28.1	-18.6 +/- 7.9
**ACS^{S52}- ABI1**	**Haddock score**	**RMSD***	E_{vdw}	E_{elec}	E_{desol}
cluster1	-142.9 +/- 1.4	13.324	-40.3 +/- 6.4	-333.4 +/- 15.1	-35.9 +/- 7.8
cluster2	-127.4 +/- 4.7	10.827	-44.0 +/- 12.2	-197.3 +/- 17.5	-43.9 +/- 6.1
cluster4	-126.4 +/- 3.7	13.260	-54.4 +/- 3.5	-130.9 +/- 36.0	-46.1 +/- 4.1
cluster9	-125.5 +/- 17.8	12.703	-40.8 +/- 12.9	-210.0 +/- 91.7	-43.8 +/- 1.7
cluster3	-118.8 +/- 7.0	13.666	-39.8 +/- 7.7	-46.0 +/- 40.9	-74.7 +/- 11.6
cluster10	-112.7 +/- 11.1	15.638	-39.7 +/- 6.0	-182.4 +/- 31.3	-36.6 +/- 6.1
cluster6	-108.9 +/- 4.4	16.156	-36.4 +/- 9.8	-140.3 +/- 36.2	-44.5 +/- 14.2
cluster5	-108.7 +/- 7.4	15.165	-37.5 +/- 5.0	-169.7 +/- 30.9	-37.3 +/- 5.5
cluster8	-106.3 +/- 9.9	14.863	-29.3 +/- 5.2	-308.1 +/- 32.8	-16.3 +/- 1.9
cluster7	-102.6 +/- 8.9	14.135	-29.8 +/- 3.2	-253.3 +/- 61.7	-22.3 +/- 16.6
**$ACS7^{S85}$- ABI1**	**Haddock score**	**RMSD***	E_{vdw}	E_{elec}	E_{desol}
cluster1	-160.4 +/- 12.2	8.673	-48.4 +/- 6.2	-330.0 +/- 63.5	-46.1 +/- 5.9
cluster2	-151.7 +/- 14.5	7.176	-41.0 +/- 11.8	-388.3 +/- 18.1	-33.2 +/- 17.3
cluster4	-135.2 +/- 22.7	9.416	-40.6 +/- 9.0	-299.7 +/- 45.8	-34.7 +/- 7.5
cluster5	-111.9 +/- 22.0	8.417	-25.6 +/- 4.1	-325.9 +/- 103.2	-21.2 +/- 13.3
cluster3	-99.2 +/- 23.1	9.398	-28.7 +/- 16.1	-295.4 +/- 31.4	-11.9 +/- 8.2
**$ACS7^{S182}$ - ABI1**	**Haddock score**	**RMSD***	E_{vdw}	E_{elec}	E_{desol}
cluster3	-134.9 +/- 7.9	9.170	-55.6 +/- 5.3	-270.4 +/- 35.6	-29.2 +/- 8.1
cluster1	-119.2 +/- 0.8	11.425	-54.1 +/- 7.2	-149.9 +/- 24.4	-40.4 +/- 8.9
cluster5	-107.0 +/- 9.8	10.018	-34.0 +/- 5.0	-270.4 +/- 58.9	-23.4 +/- 13.7
cluster2	-84.8 +/- 14.9	12.260	-18.8 +/- 3.7	-182.3 +/- 33.3	-34.3 +/- 14.5
cluster4	-76.3 +/- 5.8	16.618	-37.3 +/- 4.7	-100.2 +/- 25.4	-22.3 +/- 9.3
**$ACS7^{S183}$- ABI1**	**Haddock score**	**RMSD***	E_{vdw}	E_{elec}	E_{desol}
cluster4	-130.5 +/- 25.6	6.877	-56.3 +/- 11.2	-255.2 +/- 45.0	-27.1 +/- 11.3
cluster1	-130.3 +/- 8.8	10.618	-55.5 +/- 4.7	-153.2 +/- 21.6	-46.4 +/- 7.0
cluster3	-92.4 +/- 21.5	7.591	-24.3 +/- 7.0	-248.5 +/- 29.1	-24.1 +/- 18.8
cluster2	-80.7 +/- 5.2	11.558	-20.3 +/- 5.3	-127.3 +/- 24.6	-36.1 +/- 6.1

ACS7^{S272}-ABI1	Haddock score	RMSD*	E_{vdw}	E_{elec}	E_{desol}
cluster1	-118.8 +/- 4.1	9.090	-45.8 +/- 3.9	-135.9 +/- 23.9	-51.5 +/- 2.3
cluster3	-108.4 +/- 0.8	9.637	-37.6 +/- 4.4	-149.5 +/- 11.6	-45.8 +/- 1.7
cluster2	-108.1 +/- 7.8	10.904	-32.0 +/- 3.0	-190.2 +/- 26.1	-43.7 +/- 2.3
cluster4	-101.7 +/- 7.3	9.600	-22.8 +/- 4.8	-228.8 +/- 54.8	-39.4 +/- 7.5
cluster7	-88.4 +/- 9.2	8.390	-20.1 +/- 4.0	-206.4 +/- 33.7	-32.8 +/- 2.9
cluster6	-77.2 +/- 7.9	9.637	-27.5 +/- 3.2	-104.0 +/- 41.2	-33.2 +/- 5.6
cluster5	-76.5 +/- 6.1	15.175	-15.8 +/- 1.4	-118.9 +/- 38.7	-36.9 +/- 3.5
**ACS7^{S413}-ABI1**	**Haddock score**	**RMSD***	E_{vdw}	E_{elec}	E_{desol}
cluster2	-138.2 +/- 1.4	16.107	-47.7 +/- 8.3	-333.9 +/- 16.1	-28.0 +/- 7.8
cluster1	-118.1 +/- 1.6	13.292	-44.5 +/- 10.5	-195.8 +/- 37.2	-38.6 +/- 5.4
cluster6	-106.5 +/- 5.1	18.632	-30.0 +/- 1.7	-216.2 +/- 17.0	-33.3 +/- 4.1
cluster3	-105.5 +/- 11.2	18.164	-29.5 +/- 1.9	-220.4 +/- 47.4	-34.1 +/- 8.6
cluster4	-80.3 +/- 10.6	17.643	-21.3 +/- 4.0	-207.7 +/- 51.2	-20.7 +/- 10.7
cluster5	-78.7 +/- 11.4	14.748	-23.0 +/- 4.9	-130.1 +/- 14.9	-33.1 +/- 9.8
